# A comparative analysis of TOAST and ASCOD criteria in etiologic subtyping of acute ischemic stroke at a tertiary hospital in Tanzania

**DOI:** 10.3389/fstro.2025.1598711

**Published:** 2025-10-27

**Authors:** Mukasa Mohammed, Mabula Mussa Mabelele, Hanifa Mbithe, Ahmed Jusabani, Philip Adebayo

**Affiliations:** ^1^Department of Internal Medicine (Neurology section), Aga Khan University Medical College East Africa, Dar es Salaam, Tanzania; ^2^Aga Khan Hospital, Dar es Salaam, Tanzania; ^3^Department of Radiology and Imaging, Kilimanjaro Christian Medical Centre, Moshi, Tanzania

**Keywords:** ischemic stroke, ischemic stroke subtypes, The Trial of Org 10172 in Acute Stroke Treatment (TOAST), Atherosclerosis, Small vessel disease, Cardiac pathology, Other cause, and Dissection (ASCOD)

## Abstract

**Background:**

Etiologic subtyping of ischemic stroke is crucial for determining its treatment, prognosis, and prevention. However, data on the widely utilized TOAST and ASCOD criteria remain scarce in the East African region.

**Aims:**

The study aimed to compare the performance of the TOAST and ASCOD systems in subtyping ischemic stroke among stroke patients at a tertiary hospital in Tanzania.

**Methods:**

This was an institutional cross-sectional study. All adults (≥18 years) admitted with a diagnosis of stroke over a six-year period were selected from the registry, and their clinical details reviewed retrospectively. One hundred and thirty (130) patients with first- or second-time acute stroke (as defined by the World Health Organization) were included. Acute stroke was confirmed as ischemic by magnetic resonance imaging. For each index stroke, TOAST and ASCOD criteria were applied. The discordance and level of agreement between the approaches were assessed using McNemar's test χ^2^ (*P*-value) and Cohen's kappa coefficient (κ), respectively. The value of κ was interpreted as moderate (0.41–0.6), good (0.61–0.8), very good (0.81–0.9), or excellent (0.91–1.0). Statistical significance was set at *P* < 0.05.

**Results:**

There was no significant discordance between TOAST and the grade 1 level of evidence of ASCOD (ASCOD1) in assigning stroke to all subtypes, except for undetermined etiology χ^2^ (*P* = 0.023). Agreement between these systems was good to very good (κ = 0.601 to 0.843, *P* < 0.01) across the subtypes. TOAST and ASCOD1 failed to determine a definitive etiology in 34.6% and 48.5% of strokes, respectively. On comparing TOAST vs. combined grade of evidence 1 and 2 of ASCOD (ASCOD1,2), there was a discordance in allocation of strokes to the cardioembolic subtype χ^2^ (*P* < 0.001), and agreement was moderate (κ = 0.471, *P* = 0.001). However, the agreement across other identified subtypes was good to very good (κ = 0.601 to 0.875, *P* ≤ 0.001).

**Conclusion:**

There was a good to very good agreement between TOAST and ASCOD1 in etiologic subtyping of ischemic stroke. Further research is warranted to evaluate their consistency across diverse local settings and to explore factors influencing their performance.

## Introduction

The World Health Organization (WHO) defines stroke as the rapid onset of focal or global cerebral dysfunction lasting more than 24 h or leading to death, with no apparent cause other than vascular origin (World Health Organization. Noncommunicable Diseases, 2005). In addition to this clinical definition, the American Heart Association and the American Stroke Association emphasized a tissue-based definition of stroke, which incorporates evidence from neuroimaging or pathological examination (Coupland et al., 2017; Sacco et al., 2013).

Stroke poses a significant global health burden and is currently the second-leading cause of death worldwide, with a lifetime risk of 25% among adults (Feigin et al., 2018, 2021). Of all the strokes, ischemic stroke constitutes the majority, with its patterns and subtypes varying according to the sociodemographic and biological factors unique to a specific environment (Petty et al., 2000; Ominde, 2019; Gulli et al., 2016; White et al., 2005; Kim and Kim, 2014; Owolabi et al., 2017), underscoring the importance of context-specific stroke studies. In low- and middle-income countries, including sub-Saharan Africa, stroke is particularly burdensome because it affects a relatively younger age group with a higher morbidity and mortality compared to Europeans (Hajat et al., 2011; Akinyemi et al., 2021; Sarfo et al., 2018). Complicating this outlook is the paucity of data on stroke etiological subtypes, largely due to inadequate access to optimal investigation in these regions. In Tanzania, for example, studies (Matuja et al., 2004; Walker et al., 2011; Matuja et al., 2024) on subclassification and characterization of stroke were broad and categorized stroke into two main subcategories: hemorrhagic and ischemic. These studies also lacked detailed assessment of the etiological mechanisms involved. Another recent study in Tanzania that investigated large artery-related stroke revealed a significant prevalence of large artery occlusion (LVO) in the study population (Matuja et al., 2022). However, the study was limited by the absence of relevant confirmatory investigations, such as angiography; hence, the occlusions were reported as “presumed” rather than proven. Like other studies in the East African region (Tshikwela et al., 2015), computerized tomography (CT) scan was the predominant neuroimaging modality used to characterize ischemic strokes, although it is not the modality of choice for this purpose (Mejdoubi et al., 2017). Other advanced supportive investigations, such as carotid Doppler and magnetic resonance angiography, were not utilized to determine the underlying causative mechanism.

In the context of subtyping ischemic stroke based on the underlying etiologies, the Trial of Org 10172 in Acute Stroke Treatment (TOAST) classification system (Adams et al., 1993) remains the most widely used approach, and has served as the gold standard for epidemiological and clinical research for over three decades (Adams and Biller, 2015). TOAST subclassifies acute ischemic stroke into large artery atherosclerosis (embolus or thrombosis); cardioembolic, small vessel occlusion (lacunar); stroke of other determined cause or unusual cause; and stroke of undetermined cause, which comprises multiple identified causes, negative evaluation, or incomplete evaluation (Adams et al., 1993; Knight-Greenfield et al., 2019). With recent advancements in diagnostics, the original TOAST system underwent some modifications (Kim and Kim, 2014; Yang et al., 2019; Ay et al., 2007; Chen et al., 2012), and from it came another widely used phenotypic ischemic stroke classification system, ASCOD (A-atherosclerosis, S-small vessel disease, C-cardiac pathology, O-other cause, and D-dissection) (Amarenco et al., 2013). Unlike TOAST, which allocates a single most probable etiology and categorizes stroke with multiple probable etiologies under stroke of undetermined cause, ASCOD puts into consideration the concurrence of multiple probable etiologies in a single stroke and therefore allocates a phenotypic subtype. This criterion also assigns a graded level of certainty denoted as 1, 2, 3, 0, or 9, to each contributory etiology based on the available evidence. For instance, grade 1 indicates that the unraveled etiology is the most likely cause of the stroke event, grade 2 denotes an uncertain causal relationship, and grade 3 signifies that the etiology is unlikely to be the cause. Grades 0 or 9 are applied if evidence of causality is absent or inadequate, respectively (Amarenco et al., 2013). On the African continent the ASCOD criteria has only been used in West Africa (Sarfo et al., 2022).

Some comparative studies have shown that TOAST and ASCOD differ in their ability to assign ischemic stroke to various subtypes, with ASCOD generally reducing the proportion of undetermined categories (Arsava et al., 2017; Desai et al., 2014; Xin et al., 2019; Patel et al., 2019). Furthermore, each subtype, except the undetermined category, is affected differently by ASCOD criteria inbetween subtypes, and within the subtype depending on the grade of evidence used (Marnane et al., 2010). Most studies, however, have demonstrated a moderate to high agreement between TOAST and ASCOD across all stroke subtypes, save for the undetermined subcategory (Gökçal et al., 2017; Shang and Liu, 2012; Wolf et al., 2012).

While ischemic stroke subtyping using these subtyping systems has been shown to impact treatment, prognosis, and stroke recurrence (Walker et al., 2011; Ois et al., 2013; Adams et al., 1999; Kauw et al., 2018; Kolmos et al., 2021), rigorous subtyping is yet to be incorporated in stroke care in our region. The routine utilization of ischemic stroke subtyping systems remains limited in our context; as a result, their comparative performance is uncertain. On the African continent, to our knowledge, no published study has evaluated the performance of the widely used TOAST and ASCOD systems. This study, therefore, mainly aimed to compare the performance of the TOAST and ASCOD classification systems in etiologic subtyping of ischemic stroke at the Aga Khan Hospital, Dar es Salaam, Tanzania, by assessing both the discordance and the level of agreement between them.

## Materials and methods

### Study setting, study population, and selection procedure

This was an institutional retrospective cross-sectional study conducted at the Aga Khan Hospital, Dar es Salaam (AKHD). AKHD is an internationally accredited private tertiary hospital that provides advanced neurological services and stroke care to residents of Dar es Salaam and other adjoining cities. The 170-bed hospital is the main referral hospital, serving as the hub for other hospitals under the Aga Khan Health Services Tanzania.

**Figure 1:**
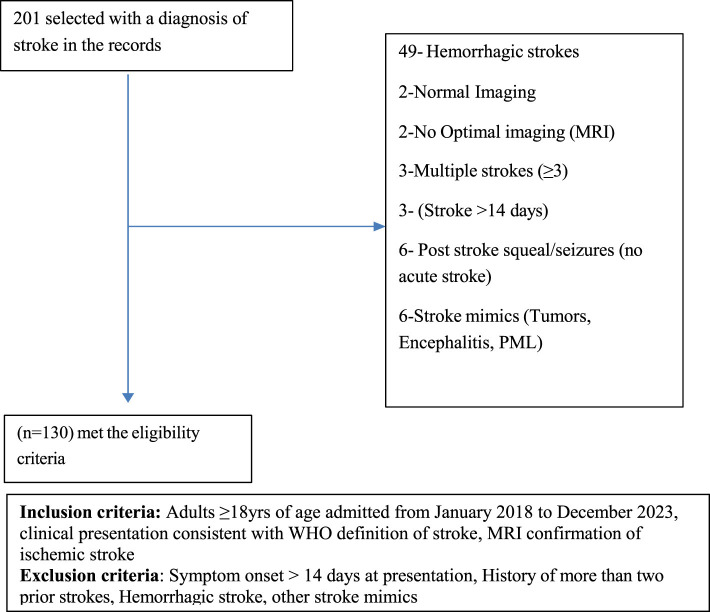
Flow chart showing the recruitment procedure.

The study population included all adult patients (aged 18 years and above) admitted to the medical department of the AKHD with a diagnosis of acute stroke between January 2018 and December 2023. A total of 201 patients were retrospectively selected through census sampling from the hospital registry. The inclusion criteria included: age of 18 years or older; a clinical diagnosis of acute stroke consistent with the WHO definition; and neuroimaging confirmation of acute ischemic stroke by magnetic resonance imaging (MRI) of the brain. Patient records were reviewed in detail, including each patient's file, hospital stroke care protocol (attached to each patient's file), and electronic medical records, to verify the consistency of the diagnosis with the clinical presentation. All cases were evaluated by a consultant neurologist or consultant physician, in accordance with the existing hospital policy, who confirmed the findings. The exclusion criteria included: presentation with stroke symptoms more than 14 days after onset; history of more than two prior strokes; and radiologically confirmed hemorrhagic stroke. This approach ensured that only patients with reliably confirmed acute ischemic stroke were included in the analysis. A total of 130 patients fulfilled the eligibility criteria and were recruited into the study. The recruitment process is demonstrated in Figure 1.

### Data collection

Data were collected from patient files, stroke care protocols, and the hospital electronic record system by the primary investigator, assisted by two research assistants who were trained medical doctors. Data were recorded on coded data collection tools containing no patient-identifying information and subsequently entered into Google sheets.

The patients' recorded demographic and clinical data (sex, age, body mass index (if recorded or stated by the clinician), ethnicity, place of residence, risk factors, comorbidities including hypertension (HTN), diabetes (DM), dyslipidemia, chronic kidney disease, hemoglobinopathy, tuberculosis (TB) and human immunodeficiency virus (HIV), history of coronary heart disease, underlying malignancy, smoking and alcohol history) were retrieved and reviewed from the hospital electronic filing system.

MRI data describing the images and stroke features (topographic pattern (location), lesion size, and vascular territory) were collected from the radiologists' reports. According to the hospital protocols that already existed, the MRI reports were written and approved by two experienced radiologists before release. The lesion size, if not reported, was measured. The recorded data were then cross-checked by another consultant radiologist who was not involved in the initial report writing. He/she also independently reviewed the images and confirmed the consistency of the collected data and the respective images. A similar process for all radiological imaging tests was followed.

Data from other supporting investigations required in stroke workup including, carotid Doppler ultrasound or magnetic resonance angiogram (MRA) reports of each patient were reviewed for the presence of atherosclerosis; further details, including laterality and severity of stenosis of the extra and intracranial arteries, were recorded in terms of approximated percentage (>50, 50–30, <30). The presence of plaque was also recorded.

Cardiac investigation (electrocardiogram (ECG) and echocardiogram) findings, as reported by an experienced cardiologist, were also reviewed for evidence of cardiac pathology. Ambulatory (Holter) ECG with reported or suspected abnormalities were also reviewed by the cardiologist who was not involved in that patient's care.

Other additional investigations performed for acute stroke, at the clinician's discretion, were also reviewed and recorded. These included coagulation profile, lipid profile, glycated hemoglobin, complete cell count, lumbar puncture, and autoimmune profile.

### Ischemic stroke subtyping

The above data, in addition to risk factor assessment, were used to assign a subtype to the ischemic stroke following the published TOAST criteria (Adams et al., 1993). According to this system, stroke was allocated to five subtypes, namely large artery atherosclerosis (LAA), small vessel occlusion (SVO), cardiac embolism (CE), stroke of other determined etiology (SOC) if a specific cause was identified, and stroke of undetermined etiology (SUC) if no cause was identified. SUC was further subdivided into stroke of undetermined etiology with a negative evaluation (NE) if no cause was found after all the necessary investigations were done, multiple etiologies (ME) if two possible etiologies were identified, and stroke of undetermined etiology with incomplete evaluation (IE) if the evaluation was not adequate for subtyping to be done. The ASCOD system was subsequently applied for alternative stroke subtyping, and the grade of evidence allocated according to the established criteria (Amarenco et al., 2013). Finally, the allocations were cross-checked and confirmed by an experienced neurologist.

### Data analysis

The data were imported and analyzed using the Jamovi software version 2.4.0. Demographic and clinical data were summarized using frequencies and percentages for categorical variables, means (with standard deviation) for continuous variables that were normally distributed, and medians and interquartile ranges for data with a skewed distribution. The proportion of each stroke etiological subtype in the TOAST and ASCOD systems was determined by frequencies (percentages). Since the data were paired, the McNemar's test was used to assess the significance of the discordance in allocation to TOAST vs. ASCOD for each subtype. The agreement between each TOAST subtype and the corresponding subtype of ASCOD with grade 1, and combined grades 1 and 2 evidence, respectively, was assessed using Cohen's kappa coefficient (κ), for which the κ-value was interpreted as moderate (0.41–0.6), good (0.61–0.8), very good (0.81–0.9), and excellent (0.91–1.0). The level of statistical significance was set at *P* < 0.05. Additional information from ASCOD was also obtained by applying various levels of evidence. According to the kappa statistic in reliability studies (Sim and Wright, 2005; Donner and Eliasziw, 1992), a sample size of 130 can detect at least moderate agreement (κ = 0.4–0.6) with balanced category prevalence, at 95% confidence and a margin of error of approximately ±0.1.

## Results

### Demographic characteristics of the study population

The study analyzed the clinical details of the selected 130 adults. All had onset of stroke symptoms within 2 weeks of presentation and a confirmed diagnosis of acute ischemic stroke by MRI of the brain. The study population was predominantly of Black ethnicity (104; 80%). The mean age was 60.8 years with a standard deviation of 13.8, and over half were above 50 years of age. Males constituted the majority (84; 64.6%), and most of the participants (98; 75.4%) resided in Dar es Salaam, Tanzania, with only a minority (16; 12.3%) coming from the neighboring island nation of Comoros and other regions, within or outside the country. Table 1 illustrates the detailed demographic characteristics.

**Table 1:** Demographic characteristics of the study population (*N* = 130).

**Demographic characteristic**	***n* (%)**
**Age (years), mean** +**/-SD; 60.8**+**/- (13.8)**	
≤ 39	6 (4.6)
40–59	49 (37.7)
≥60	75 (57.7)
**Gender**	
Female	46 (35.4)
Male	84 (64.6)
**Residence**	
Dar es Salaam	98 (75.4)
Comoros	16 (12.3)
Other	16 (12.3)
**Ethnicity**	
Asian	19 (14.6)
Black	104 (80.0)
Caucasian	2 (1.5)
Unknown	5 (3.8)

### Clinical characteristics

Of the 130 patients, 103 (79.2%) had a stroke for the first time, and 27 (20.8%) had it for the second time. No patient had more than two previous strokes. All patients had undergone daily evaluation by a senior consultant physician (consultant internal medicine physician in the neurology unit), a consultant neurologist, or both, who had reaffirmed the clinical findings. The majority (104; 80%) were under a neurologist as the primary care specialist. Hypertension (99; 76.2%) and diabetes (38; 29.7%) were the most common risk factors in the study population. Twenty-two (17.2%) had a history of being on antiplatelet medications before the event. Other patients' comorbidities are shown in Table 2.

**Table 2:** Clinical characteristics, comorbidity, and risk factors of the study population (*N* = 130).

**Clinical characteristics and risk factors**	***n* (%)**
**Primary caring medical specialist**	
Neurologist	104 (80.0)
Non neurologist physician only	22 (16.9)
Both above	4 (3.1)
**Stroke history**	
First ever stroke	103 (79.2)
Repeat (2nd) stroke	27 (20.8)
**Risk factors/comorbidity**	
Hypertension	99 (76.2)
Diabetes	38 (29.7)
Obesity	22 (17.2)
Chronic kidney disease	10 (7.7)
Dyslipidemia	8 (6.2)
IHD (Remote history)	8 (6.2)
IHD in (Recent: <4weeks)	2 (1.5)
Previous TIA	7 (5.4)
HIV	6 (4.6)
Haemoglobinopathy	1 (0.8)
Tuberculosis	2 (1.5)
Cancer	3 (2.3)
Cervical spondylosis	3 (2.3)
Migraine	3 (2.3)
Atrial fibrillation	2 (1.5)
Peripheral vascular disease	2 (1.5)
OSA	3 (2.3)
Alcohol	23 (17.7)
Smoking	18 (13.8)
**Preventive therapy**	
Statins	20 (15.4)
Anticoagulants	13 (10.0)
Antiplatelets	22 (17.2)

IHD, ischemic heart disease; HIV, human immunodeficiency virus; OSA, obstructive sleep apnea; TIA, transient ischemic attack.

### Investigations done

All patients underwent brain MRI, including diffusion-weighted imaging. Further intracranial and extracranial evaluation with MRA was performed in 92 (70.8%) of patients, CT angiography in 22 (16%), and carotid Doppler ultrasound in 113 (86.9%). Notably, brain CT scan without contrast had also been performed in a considerable proportion of patients (86; 66.1%), primarily during the initial evaluation. Cardiac evaluation with a 12-lead ECG or ambulatory ECG (Holter monitoring) was performed in 125 (96.2%) patients, in addition to transthoracic echocardiogram for comprehensive cardiac assessment. Specified imaging investigations, including magnetic resonance venography (MRV) and lower limb arterial venous Doppler ultrasound were performed in one to five (3–4%) patients, where intravascular thrombosis was suspected. Table 3 summarizes the imaging investigations performed.

**Table 3:** Imaging investigations.

**Imaging investigation**	***n* (%)**
Brain MRI	130 (100)
Extra cranial/intracranial MRA	92 (70.8)
Brain CT scan (without contrast)	86 (66.1)
CT angiography	22 (16.9)
Carotid Doppler ultrasound	113 (86.9)
Transthoracic echocardiogram	123 (94.6)
12 lead EGG	125 (96.2)
24-h Holter ECG monitor	41 (31.5)
48-h Holter ECG monitor	2 (1.5)
**Other imaging tests (based on further assessment)**	
Arterial venous Doppler ultrasound (lower limbs)	5 (3.8)
CT PA	1 (0.7)
CT abdomen for malignancy	2 (1.5)
Brain MRV	4 (3.1)
Pelvic ultrasound	1 (0.7)

Baseline and disease-specific laboratory investigations based on etiological suspicion were conducted in all patients. The most frequently performed baseline blood test was a complete blood cell count, undertaken in 126 patients (96.7%), of which leukocytosis and thrombocytosis were found in 11 and 1 patients, respectively. Elevated glycated hemoglobin level above 7% was found in 34 (33%) of the 103 patients on whom it was performed regardless of their diabetic status. The baseline and laboratory investigations performed are summarized in Table 4.

**Table 4:** Laboratory investigations.

**Laboratory test**	***n* (%)**	**Abnormal (%)**
**Complete blood cell count**	126 (96.9)	
Hemoglobin		11/126 (8.7)
WBC		18/126 (14.3)
Absolute eosinophil count		3/126 (2.4)
Platelets count		1/126 (0.8)
**Markers of inflammation/infection**		
ESR	19 (14.7)	14/19 (73.7)
C-reactive protein	85 (65.4)	21/85 (24.7)
Procalcitonin	7 (5.4)	3/7 (42.9)
D-dimer	29 (22.7)	21/29 (72.4)
**Lipids**		
Cholesterol	114 (87.7)	43/114 (37.7)
Triglycerides	113 (86.9)	37/113 (32.7)
HDL-C	111 (85.4)	49/111 (44.1)
**Cardiac tests**		
PROBNP	26 (20.2)	18 (69.2)
Troponin	77 (59.2)	40/77 (51.9)
**Glycated hemoglobin**	103 (79.8)	34/103 (33.0)
**Other**		
CSF analysis	2 (1.5)	1/2 (50.0)
ANA	5 (3.8)	0/5 (0.0)
C-ANCA and P-ANCA	3 (2.3)	0/5 (0.0)
Antiphospholipid antibodies	2 (1.5)	0/5 (0.0)
Protein C and S and antithrombin III levels	2 (2.3)	1/2 (50.0)
Homocysteine levels	2 (1.5)	0/2 (0.0)
Blood cultures	3 (2.3)	2/3 (66.6)

**Abnormal cut-offs**: WBC (white blood cell) > 12 x 10^9^/μl; hemoglobin level > 16g/dl; absolute eosinophil count > 0.5 x 10^9^/μl; platelet count > 800x 10^9^/μl; C-reactive protein > 50mg/dl; procalcitonin > 0.5 μg/l; D-dimer > (1μg/ml); cholesterol ≥ 5.2 mmol/L; HDL-C ≤ 1.03 mmol/L; triglycerides ≥ 1.7 mmol/L; LDL-C ≥ 3.4 mmol/L; ANA (antinuclear antibodies), C/P-ANCA (cytoplasmic/perinuclear antineutrophil cytoplasmic antibody), glycated hemoglobin > 7%.

Other tests conducted included lumbar puncture and cerebral spinal fluid (CSF) analysis performed on two patients based on clinical and radiological suspicion of TB vasculitis or intracranial infection. A positive result for *Mycobacteria tuberculosis* was found on GeneXpert in one of the two patients. Significant low protein C and S levels were found in only one of the two patients on follow-up testing. Other tests (ANA, P-ANCA, C-ANCA, and antiphospholipid antibodies) did not reveal any positive results in the patients on which they were performed. Blood cultures were also done on three patients suspected of concurrent sepsis; two yielded positive results, one for *Klebsiella pneumoniae* and the other for coagulase-negative cocci.

### Stroke characteristics

#### Presenting symptoms

**Figure 2:**
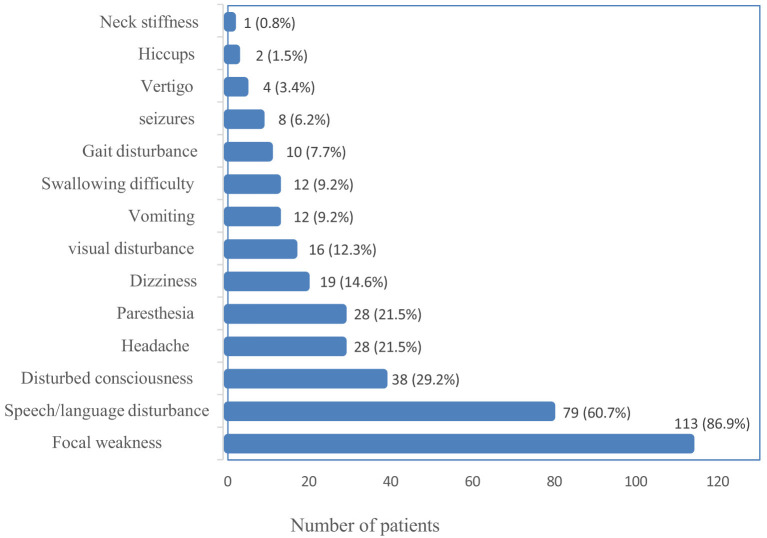
Bar graph of presenting symptoms.

The most common presentation of ischemic stroke in the study population was focal weakness (paresis) of either the face or the lower or upper limb(s), which occurred in 113 (86.9%), followed by speech disturbance in 79 (60.7%). One patient presented with neck stiffness, the least common presenting symptom. Figure 2 summarizes the frequencies of the presenting symptoms.

#### Lesion characteristics

In the majority of patients, the lesion was an ischemic infarct 124 (95.4%), and in six cases (4.6%) it was ischemic with some hemorrhagic transformation. Based on anatomical territory, stroke occurred in a single or multi-territory in 79 (60.8%) and 51 (39.2%) patients, respectively. However, based on vascular territory, the infarct involved the anterior circulation in 74 patients (56.9%), the posterior circulation in 31 (23.8%), and both in 25 (19.2%), as shown in Table 5.

**Table 5:** Ischemic lesion characteristics.

**Infarct nature**	***n* (%)**
Ischemic	124 (95.4)
Infarct with hemorrhagic transformation	6 (4.6)
**Anatomical distribution**	
Multi-territory	51 (39.2)
Single territory	79 (60.8)
**Vascular territory**	
Anterior circulation	74 (56.9)
Posterior circulation	31 (23.8)
Both	25 (19.2)

### Frequencies of TOAST subtypes

**Figure 3:**
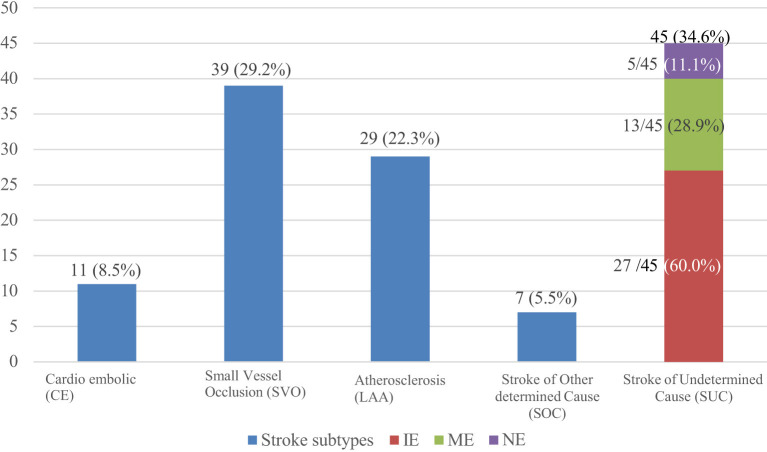
Graph showing the frequencies of ischemic stroke subtypes according to TOAST.

TOAST allocated the largest proportion 45 (34.6%) of strokes under stroke of undetermined cause (SUC), and the smallest, 7 (5.5%) under stroke of other determined cause (SOC). For stroke where a single etiology could be identified, the most frequent etiology 39 (29.2%) was small vessel occlusion, followed by large artery atherosclerosis 29 (22.3%) and then cardioembolic 11 (8.5%). Of the 45 patients under the SUC subtype, incomplete evaluation (IE) was the most frequent category (27/45; 60%), and the least frequent was those who had a negative evaluation (5/45; 11.1%) (Figure 3).

### Frequencies of ASCOD subtypes (phenotypes)

#### Grade 1 level of evidence

When grade 1 level of evidence (definite cause) was used, as illustrated in Table 6, S1 was the most frequent phenotype 31 (23.8%), followed by A1: 28 (21.5%) and C1: 13 (10.0%). No patient was identified in D1. As the grade of evidence changed from 1 to 3 through 2, there was a decrease in the number of patients and frequency in A1 to A2, S1 to S2, and O1 to O2, from 28 (21.5%) to three (2.3%), 31 (23.8%) to 12 (9.2%), and nine (6.9%) to 0 (0.0%), respectively. Conversely, there was an increase from C1 to C2 (10% to 17%). Grade 9 (insufficient workup for subtyping to be done) was most frequent in O 22 (16.9%), and zero (0.0%) in S.

**Table 6:** ASCOD phenotypes.

**Phenotypes**	**ASCOD Grade**				
	**1**	**2**	**3**	**0**	**9**
	***n*** **(%)**	***n*** **(%)**	***n*** **(%)**	***n*** **(%)**	***n*** **(%)**
A	28 (21.5)	3 (2.3)	30 (23.1)	58 (44.6)	11 (8.5)
S	31 (23.8)	12 (9.2)	30 (23.1)	57 (43.8)	0 (0.0)
C	13 (10.0)	17 (13.3)	13 (10.0)	81 (63.3)	6 (4.6)
O	9 (6.9)	0 (0.0)	6 (4.6)	93 (71.5)	22 (16.9)
D	0 (0.0)	0 (0.0)	2 (1.5)	114 (87.7)	14 (10.8)

#### ASCOD phenotypes with combined grades of evidence

When the combined level of evidence grades 1 and 2 (definite and likely cause, respectively) was applied, as shown in Table 7, there was an overall increase in the number of strokes in A (31/130; 23.8%), S (43/130; 33.1%), and C (30/130; 23.1%) as more phenotypes were identified. This increase did not occur in O or D, as O2 and D2 had no strokes assigned to that subtype. Hence, no additional patients were added by the combined approach. Considering the combination of evidence grades 1, 2, and 3, whereby the presence of disease was identified regardless of causality, there was an increase in the number of strokes across all phenotypes.

**Table 7:** Stroke subtypes according to ASCOD with combined levels of evidence and other extra information.

**Phenotypes**	**ASCOD grade**						**Disease present**
	**1**	**2**	**3**	**0**	**9**	**(1 and 2)**	**(1, 2, and 3)**
	***n*** **(%)**	***n*** **(%)**	***n*** **(%)**	***n*** **(%)**	***n*** **(%)**	***n*** **(%)**	***n*** **(%)**
A	28 (21.5)	3 (2.3)	30 (23.1)	58 (44.6)	11 (8.5)	31 (23.8)	61 (46.9)
S	31 (23.8)	12 (9.2)	30 (23.1)	57 (43.8)	0 (0.0)	43 (33.1)	73 (56.2)
C	13 (10.0)	17 (13.3)	13 (10.0)	81 (63.3)	6 (4.6)	30 (23.1)	43 (33.1)
O	9 (6.9)	0 (0.0)	6 (4.6)	93 (71.5)	22 (16.9)	9 (6.9%)	15 (11.5)
D	0 (0.0)	0 (0.0)	2 (1.5)	114 (87.7)	14 (10.8)	0 (0.0)	2(1.5)
**Extra**							
ASCODX	63 (48.5)						
ASCODN				4 (3.1)			
ASCODI					0 (0.0)		
ASCODY						50 (38.5)	
ASCODZ							16 (12.3)

ASCODX, No grade 1 evidence for a single etiology (combinations: A2/A3/A0/A9, S2/S3/S0/S9, C2/C3/C0/C9, O2/O3/O0/O9, D2D3D0D9). ASCODN, No disease at any grade of evidence was identified/all necessary investigations were negative for disease (combinations: A0S0C0O0D0). ASCODI, insufficient workup for any form of subtyping to be done (combinations, A9S9C9O9D9). ASCODY, No grade 2 or 3 evidence (combinations: A3/A0/A9, S3/S0/S9, C3/C0/C9, O3/O0/O9, D3/D0/D9). ASCODZ, No grade 1, 2, or 3 evidence/no etiology identified (combination: A0/A9, S0/S9, C0/C9, O0/O9, D0/D9).

#### Extra information from ASCOD combinations

Additional information (Table 7) that could be drawn from ASCOD on further combinations included strokes whose etiology could not be identified when grade 1 evidence was used (ASCODX, 63/130; 48.5%), and when combined grades 1 and 2 were used (ASCODY, 50/130; 38.5%), as well as instances where no disease was found (ASCOZ, 16/130; 12.3%) and where exhaustive investigation yielded no single etiology (ASCODN, 4/130; 3.1%). ASCODI, where failure to identify the presence of any disease was purely due to lack of minimum relevant workup, was not identified in any patient.

### Comparison of TOAST and ASCOD criteria

#### TOAST vs. ASCOD (1)

Both systems assigned most strokes under unidentified etiology ASCOD (1), at 45 (34.6%) compared to 34.6% by TOAST. For identified single etiology, both systems allocated most patients under the small vessel disease (SVO/S1) subtype, followed by atherosclerotic disease (LAA/A1), cardioembolic (CE/C1), and, finally, other identified causes (O; O and D in case of ASCOD). Compared to TOAST across all subtypes, ASCOD (1) decreased the LAA/A1 and SVO/S1 assignments by one and seven strokes, respectively. Conversely, it increased the allocations to CE/C1 and other (SOC/O) etiology by two strokes in each. However, in both scenarios, there was no statistically significant discordance in the allocation. The level of agreement between the two systems across all allocations was good to very good (κ= 0.601–0.843) and was statistically significant, except for the undetermined category, which showed only moderate agreement (κ= 0.441) and a statistically significant discordance χ^2^ (*P* = 0.023). Table 8 illustrates the comparison of TOAST and ASCOD (1).

**Table 8:** Comparison of TOAST and ASCOD 1.

**Subtypes**	**TOAST**	**ASCOD (1)**	**TOAST vs. ASCOD (1)**	
	***n*** **(%)**	***n*** **(%)**	χ^2^ **(*****P*****-value)**	κ **(*****P*****-value)**
LAA/A1	29 (22.3)	28 (21.5)	0.705	0.843 (<0.001)
SVO/S1	38 (29.2)	31 (23.8)	0.052	0.744 (<0.001)
CE/C1	11 (8.5)	13 (10.0)	0.414	0.725 (<0.001)
SOD/(O+ D)1	7 (5.4)	9 (6.9)	0.414	0.601 (<0.01)
SUD/ASCODX	45 (34.6)	63 (48.5)	0.023	0.441 (<0.01)

O+D, Combined O1 and D1; ASCODX, No grade 1 evidence for a single etiology (combinations: A2/A3/A0/A9, S2/S3/S0/S9, C2/C3/C0/C9, O2/O3/O0/O9, D2D3D0D9); κ, Cohen kappa coefficient.

#### TOAST and combined ASCOD (1,2)

Compared to TOAST, combined grades of evidence 1 and 2 of ASCOD increased the number of strokes allocated to all subtypes. The largest increase was 19 (14.6%) and occurred in the CE/C2 subtype. The discordance in allocation to this subtype was also different from the rest, as indicated by a statistically significant difference (*p* < 0.001), and there was moderate agreement (κ = 0.471) between the two systems. However, the agreement in other subtypes [LAA/A1,2, SVO/S1,2, CE/C1,2, and SOD/(O+D) (1,2)] was good to very good (κ = 0.601–0.875). The details are illustrated in Table 9.

**Table 9:** Comparison of TOAST and ASCOD (1,2).

**Subtypes**	**TOAST**	**ASCOD (1,2)**	**TOAST vs. ASCOD (1,2)**	
	***n*** **(%)**	***n*** **(%)**	χ^2^ **(*****P*****-value)**	κ **(*****P*****-value)**
LAA/A1,2	29 (22.3)	31 (23.8)	0.480	0.827 (0.001)
SVO/S1,2	38 (29.2)	43 (33.1)	0.059	0.875 (0.001)
CE/C1,2	11 (8.5)	30 (23.1)	<0.001	0.471 (0.001)
SOD/(O+ D)1,2	7 (5.4)	9 (6.9)	0.414	0.601 (<0.001)
SUD/ASCODY	45 (34.6)	50 (38.5)	<0.001	0.520 (<0.001)

ASCODY, No grade 2 or 3 evidence (combinations: A3/A0/A9, S3/S0/S9, C3/C0/C9, O3/O0/O9, D3/D0/D9).

## Discussion

In this study, most participants (64.6%) were male, predominantly of Black ethnicity (80%), and resided in Dar es Salaam, Tanzania's commercial capital. The average age was 60.8 years, and more than half (57.7%) were aged above 60. Hypertension and diabetes were the most common risk factors. These demographic features closely align with findings from the SIREN study of West Africans in Ghana and Nigeria (Sarfo et al., 2022), and with the SLESS study in the UK (Gulli et al., 2016), where the mean age for Black patients was 65 years, and hypertension and diabetes were also the leading risk factors, at 83% and 40%, respectively. Similarly, a study conducted in northwestern Tanzania reported comparable characteristics among ischemic stroke patients, with a mean age of 67 ± 15 years and 85% having premorbid hypertension (Matuja et al., 2024).

Clinically, most strokes (86%) presented with focal weakness (paresis). This is consistent with other studies (Matuja et al., 2024; Rathore et al., 2002), where paresis occurred in up to 80% of ischemic strokes overall, and up to 85% among Black participants (Rathore et al., 2002). In addition, most strokes occurred in the anterior circulation territory in 74 cases (56.9%) and in the posterior circulation in 31 (23.8%). The predominance of anterior circulation ischemia aligns with the results of other studies (Go, 2015; Tao et al., 2012).

In tandem with other research (Desai et al., 2014; Xin et al., 2019; Patel et al., 2019; Gökçal et al., 2017), our findings demonstrated good agreement between ASCOD (1) and TOAST, with weaker agreement observed when ASCOD grades 1 and 2 were combined [ASCOD (1,2)]. In this study, however, the decline in agreement was much more pronounced in the cardioembolic subtype (C 1,2), where there was also statistically significant discordance between the two systems. This may partly be explained by the study's relatively limited scope of cardiac evaluation. The overall reduction in agreement when moving from grade 1 evidence to combined grades likely reflects the lower probability of causality when incorporating lower levels of evidence. Unlike most studies (Arsava et al., 2017; Desai et al., 2014; Xin et al., 2019; Patel et al., 2019), where the application of ASCOD at all grades of evidence (1, 1 + 2, and 1 + 2 + 3) reduced assignments to stroke of undetermined cause compared to TOAST; this occurred only in the combined grade (1 + 2) in our study.

In the index study, the TOAST system generally, and unexpectedly, allocated fewer strokes to the undetermined category than ASCOD. A similar finding was reported in a survey from China, where ASCOD1 did not reduce the allocation to undetermined etiologies (Shang and Liu, 2012). That study also faced similar limitations in cardiac evaluation whereby transesophageal echocardiogram was not performed in any patient, and only 81.4% underwent diffuse-weighted MRI of the brain. It was also a single-center study. In our setting, the finding may partly relate to the historical fact that TOAST was developed in the 1990s when advanced investigations were less available, and CT scanning alone was often utilized in subtyping. Although this study only included MRI-confirmed ischemic strokes, other previously mentioned limitations may have impacted on the overall comparative performance of these systems.

Another important finding was the high proportion of strokes of undetermined cause, which was the most frequent category in our cohort, with the majority attributed to insufficient evaluation. This is comparable to another study, where undetermined etiology constituted the largest subtype by TOAST and ASCO (Gökçal et al., 2017). However, TOAST in our study allocated more patients to this category (SUC) than the older version of ASCOD (ASCO) used in that study. Additionally, that study was limited to ischemic stroke in young patients. Notably, unlike some studies (Desai et al., 2014; Xin et al., 2019), which considered only patients with minor stroke features while performing subtyping and comparison, our study did not exclude critically ill patients, including those admitted to an intensive care unit (ICU), whether on mechanical ventilation or receiving palliative care. This posed challenges in completing imaging investigations, particularly in the ICU setting, and raised questions about the relevance of exhaustive testing in end-of-life situations. A similar challenge was reported in another large community-based study in north Dublin, where not all tests could be done in very severe disease or were deemed inappropriate in terminal care (Marnane et al., 2010). In that study, ASCOD also failed to substantially reduce the proportion of strokes allocated to the undetermined category, a finding consistent with the index study.

## Study strengths and limitations

This study is the first to compare the TOAST and ASCOD systems in the African context. It is also the first to have utilized the ASCOD system in Tanzania and the broader regions of East, Central, and Southern Africa, and the second on the continent after the SIREN study conducted in West Africa to apply the ASCOD system. In addition, all patients included had undergone MRI of the brain, and in several instances comprehensive diagnostic workup was performed when clinically indicated.

However, the study also encountered several limitations. It was a single-center, retrospective study conducted in a private health facility that may have better access to diagnostic tools and resources than public or rural facilities. As a result, the findings may not be fully generalizable to the broader Tanzanian population or resource-poorer settings.

Notably, extended cardiac monitoring beyond 48 h and a transesophageal echocardiogram, as required in some instances in ASCOD, were unavailable to consolidate the cardiac workup. This may have resulted in an underestimation of the number of patients allocated to the cardioembolic etiology and hence affected the performances in this category.

## Conclusion

When ASCOD was applied at grade 1 level of evidence, both ASCOD and TOAST demonstrated good to very good agreement in subtyping acute ischemic stroke in our setting, with no significant discordance between the two approaches. Furthermore, the ASCOD system did not significantly reduce the number of ischemic strokes allocated to the stroke of undetermined etiology category compared to the general trend observed in previous studies.

This study provides valuable insight into the use of TOAST and ASCOD systems in ischemic stroke subtyping in the Tanzanian context, highlighting challenges in etiological classification, particularly in retrospective analyses. Further research is warranted to examine the consistency and factors influencing the performance of these systems in the local context, including the availability of diagnostic tools such as MRI. Prospective, multicenter study designs may help overcome limitations related to incomplete evaluation and reliance on retrospective data. ASCOD, which evaluates concurrent etiologies in greater detail, may enhance comprehensive stroke prevention and also merits further study in this setting.

## Data Availability

The raw data supporting the conclusions of this article will be made available by the authors, without undue reservation.
