# Supplementary material for: A comparative analysis of TOAST and ASCOD criteria in etiologic subtyping of acute ischemic stroke at a tertiary hospital in Tanzania
Source: Front Stroke. 2025 Oct 27;4:1598711. doi: 10.3389/fstro.2025.1598711 (PMC12802723; doi:10.3389/fstro.2025.1598711)

# **APPENDICES**

## APPENDIX I: TOAST Classification ([Adams et al., 1993](#_ENREF_1))

LAA

Clinical evidence of cortical, subcortical, brain stem, or cerebellar dysfunction (A history of intermittent claudication, (TIAs)

in the same vascular territory, a carotid bruit, or diminished pulses helps support the clinical diagnosis),

Associated with more than 50% stenosis or occlusion in an extracranial or intracranial vessel in the distribution of an infarct larger than 1 by 15mm on CT or MRI.

In the absence of evidence of pathology on arterial studies, or any other study or history that suggests that another mechanism is possible.

Diagnosis cannot be made if duplex or arteriographic studies are normal or show only minimal changes.

SVO

A lacunar syndrome (pure motor, sensorimotor, pure sensory, ataxia hemiparesis, dysarthria clumsy hand) with normal CT or MRI or a lesion smaller than 15mm on CT or MRI in the territories supplied by small-vessel penetrators. A history of DM or HTN supports the clinical diagnosis.

Large-artery and cardiac sources must be excluded.

CE

Clinical evidence of cortical, subcortical, brain stem, or cerebellar dysfunction with a lesion size larger than 1.5 cm on CT or MRI and the presence of at least **one high-risk** (e.g., atrial fibrillation or mechanical heart valve,) or **medium-risk cardiac pathology** (e.g., lone atrial fibrillation or patent foramen ovale) on diagnostic studies, electrocardiogram, rhythm strip, 24-h cardiac monitoring, and transthoracic or transesophageal echocardiography. Evidence of strokes in more than one vascular territory or of systemic emboli supports the diagnosis. Other categories (large artery, small artery) must be excluded.


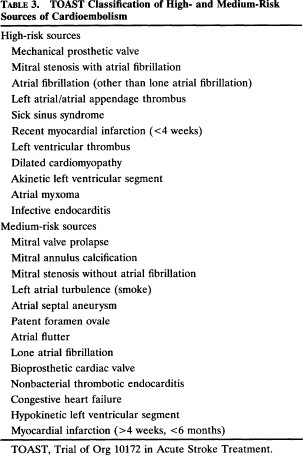


SOC

Stroke caused by nonatherosclerotic vasculopathies, hypercoagulable states, or hematologic disorders and other rare causes of stroke after diagnostic testing. Other categories must be excluded

**SUC: (**NE, ME, IE)

**LAA**: Large Artery Atherosclerosis

**SVO:** Small vessel Occlusion

**CE:** Cardio embolic

**SOC:** Stroke caused by Other determined Cause

**SUC:** Stroke of Undetermined Cause (NE- Negative evaluation, ME-Multiple Etiology, Stroke of un-determined Etiology with incomplete evaluation I.E)

Definitions for use in a multicenter clinical trial. TOAST. Trial of Org 10172 in Acute Stroke Treatment. *Stroke, 24*(1), 35-41. doi:10.1161/01.str.24.1.3

## APPENDIX II: ASCOD Classification ([Amarenco et al., 2013](#_ENREF_1))

**Grading of ASCOD phenotypes.**

1. If the disease is present and can potentially be a cause

2. If the disease is present, but the causal link is uncertain

3. If the disease is present, but the causal link is unlikely

0. If the disease is absent

9. If the workup is insufficient to grade the disease

**Atherothrombosis (A), Small vessel disease (S), Cardiac pathology (C), Other causes (O), Dissection (D)**


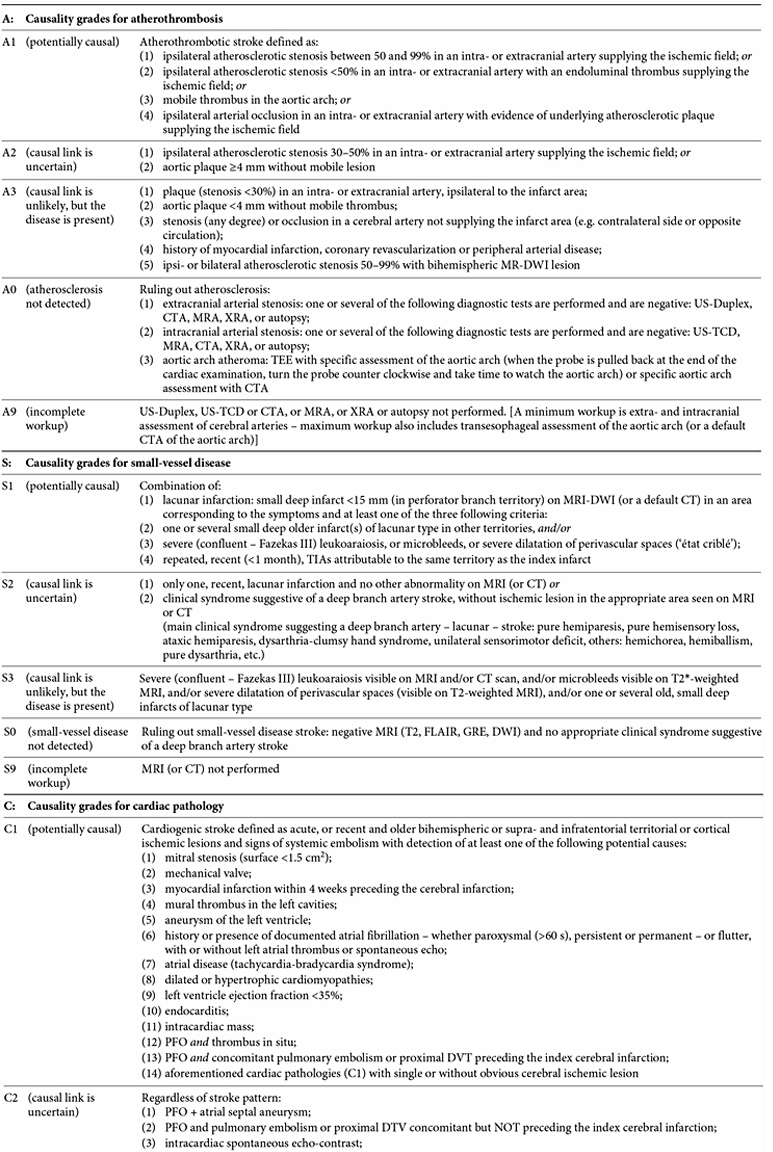


## APPENDIX III: Data collection tool


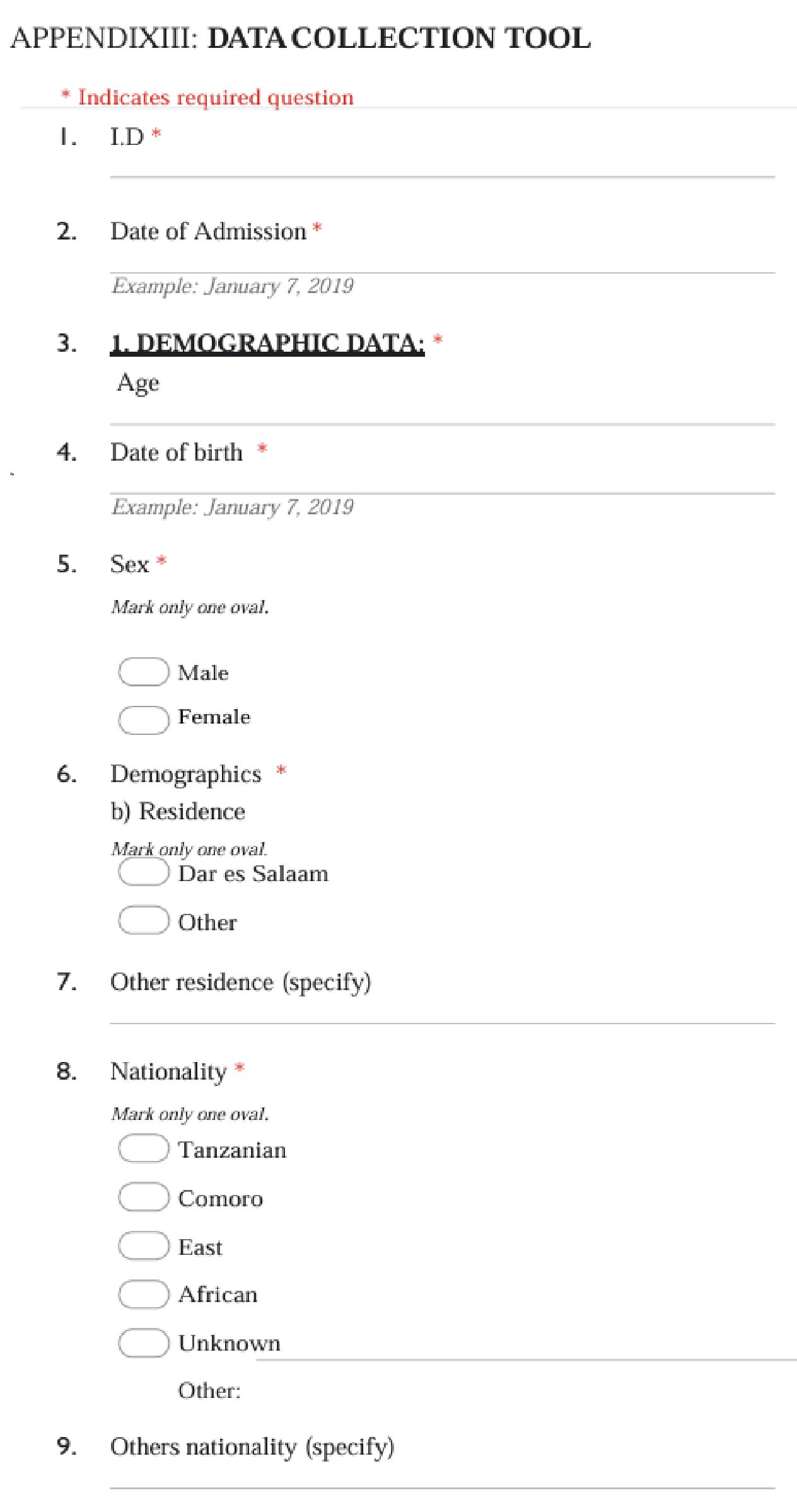

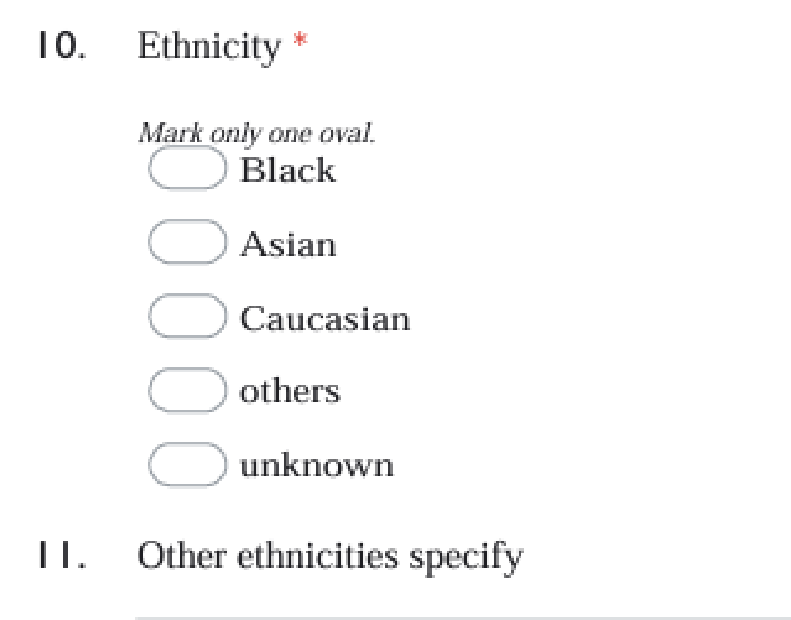

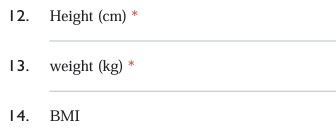

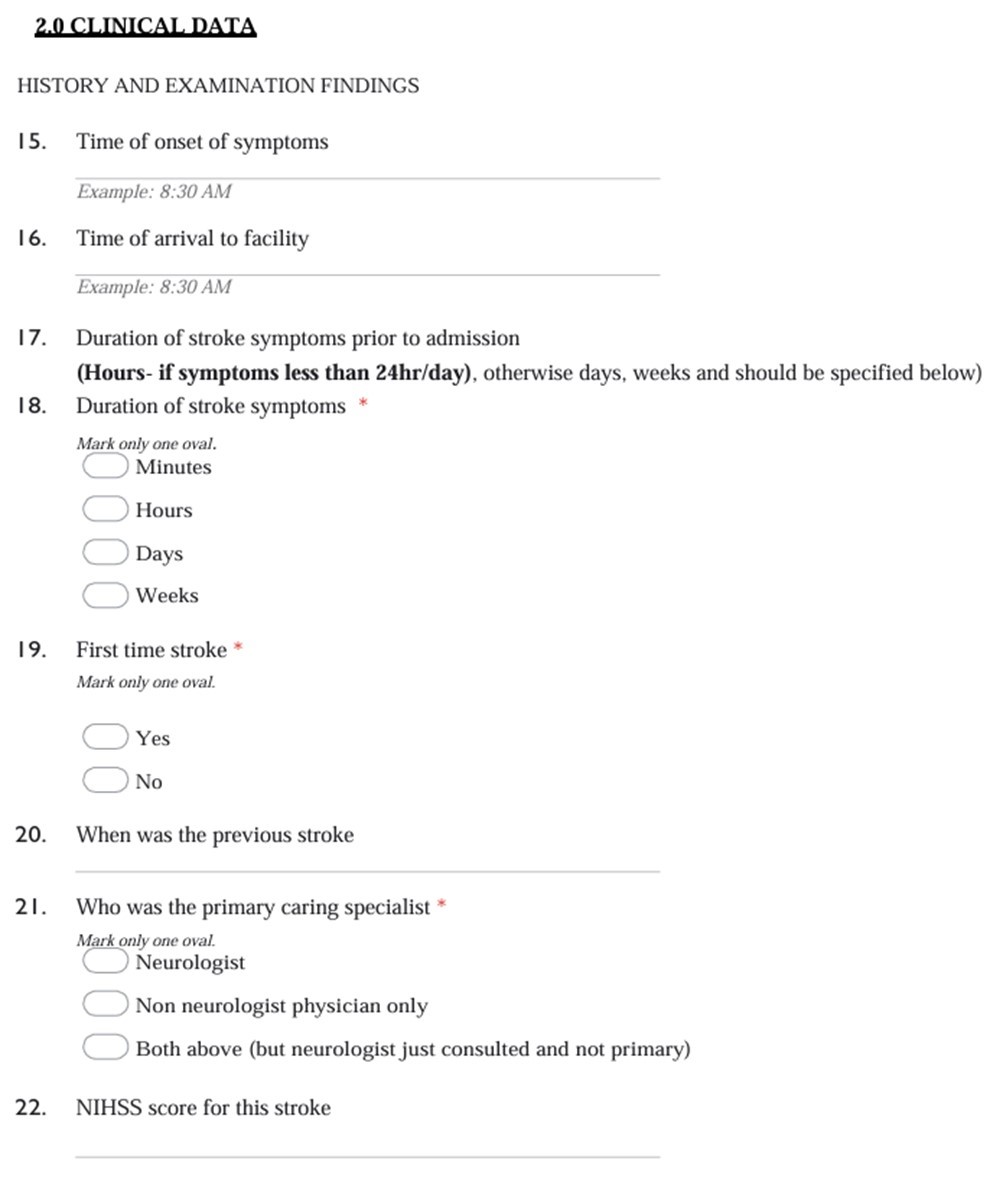

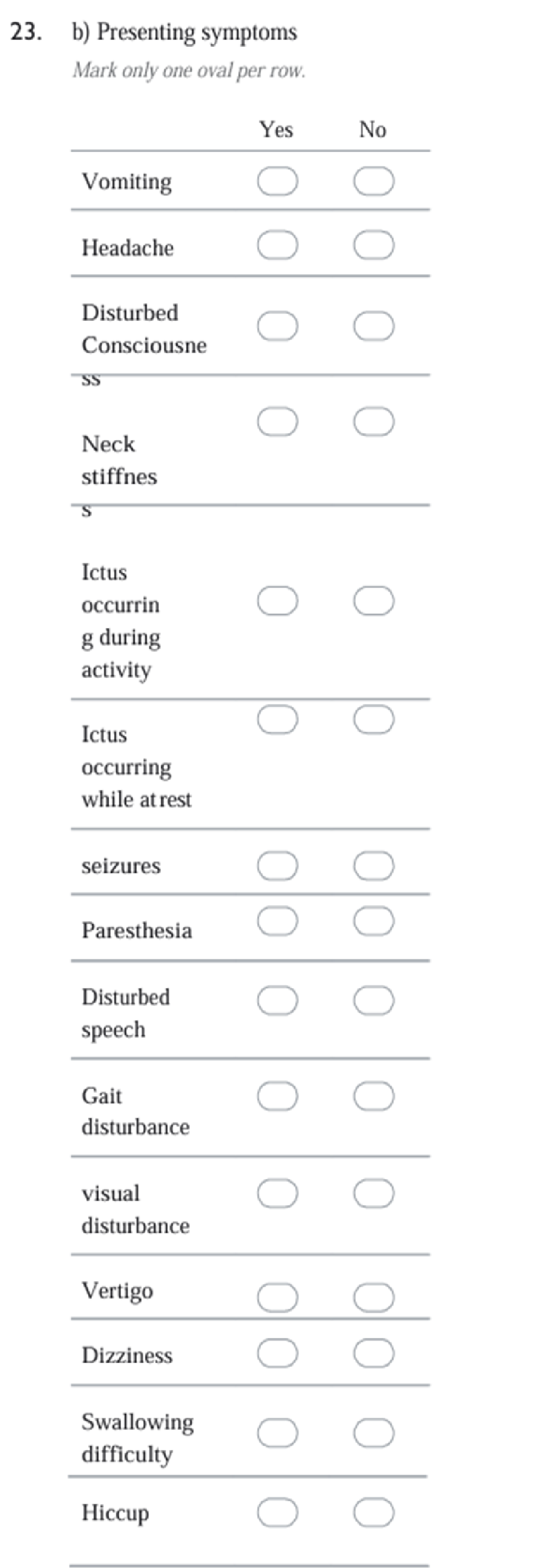

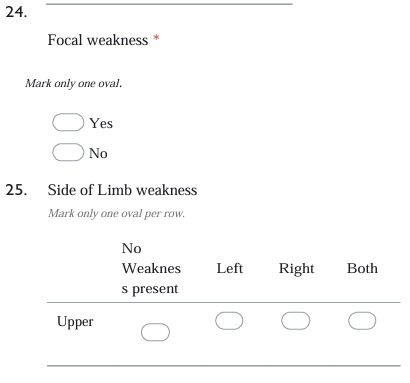

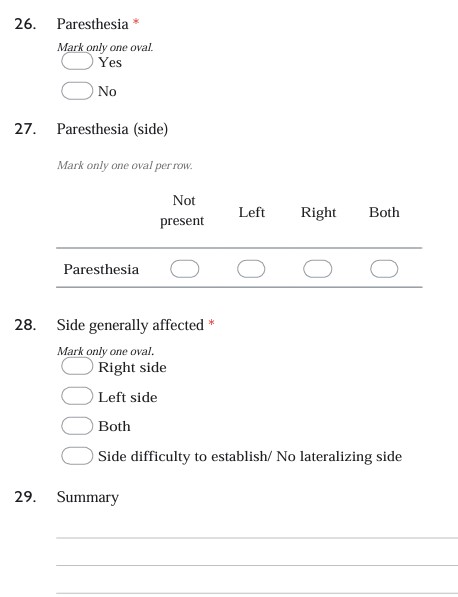

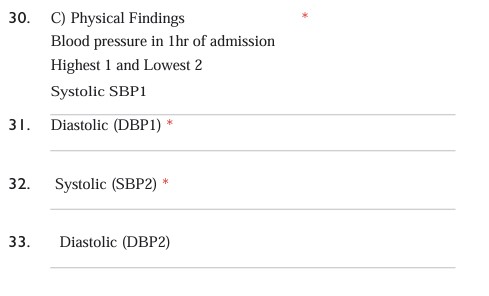

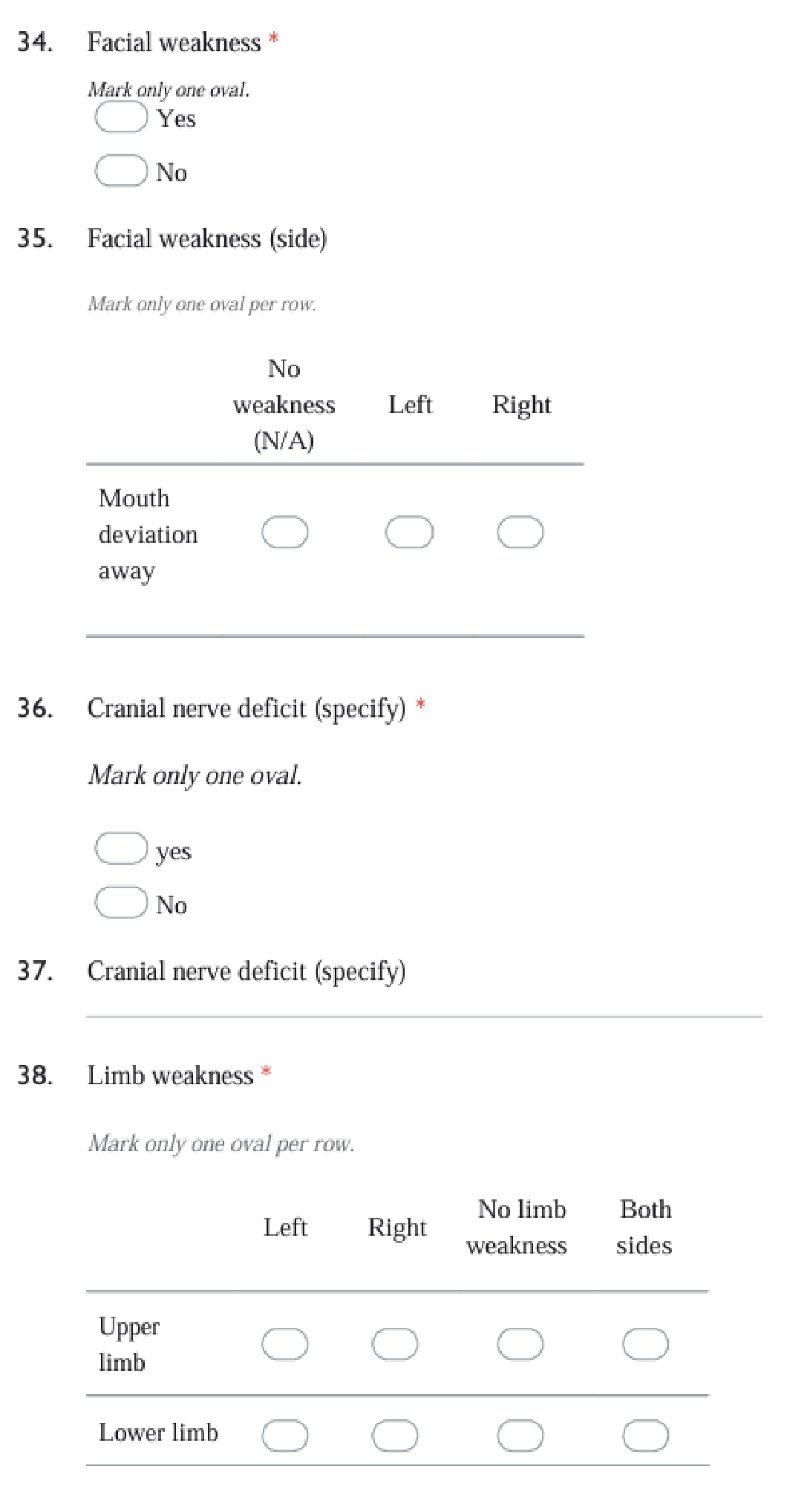

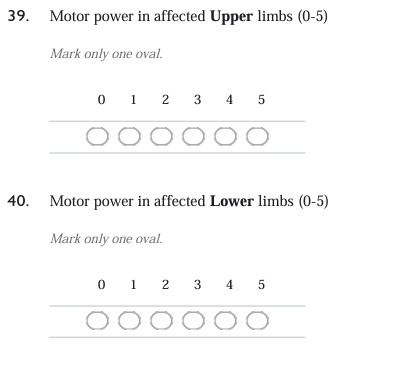

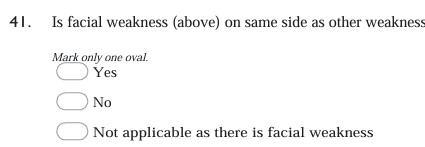


Other finding on exam


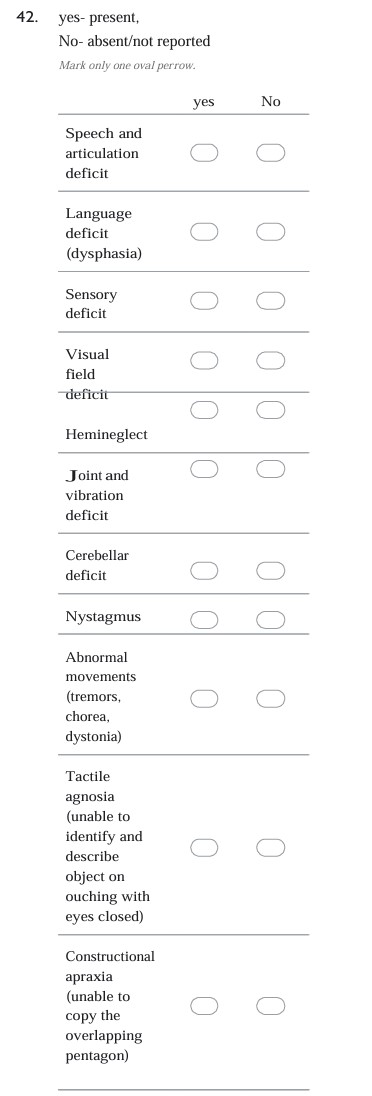


43

. Any neurological sign not stated above

(

example type of gait, gag reflex), pronator drift

……………………………………………………………


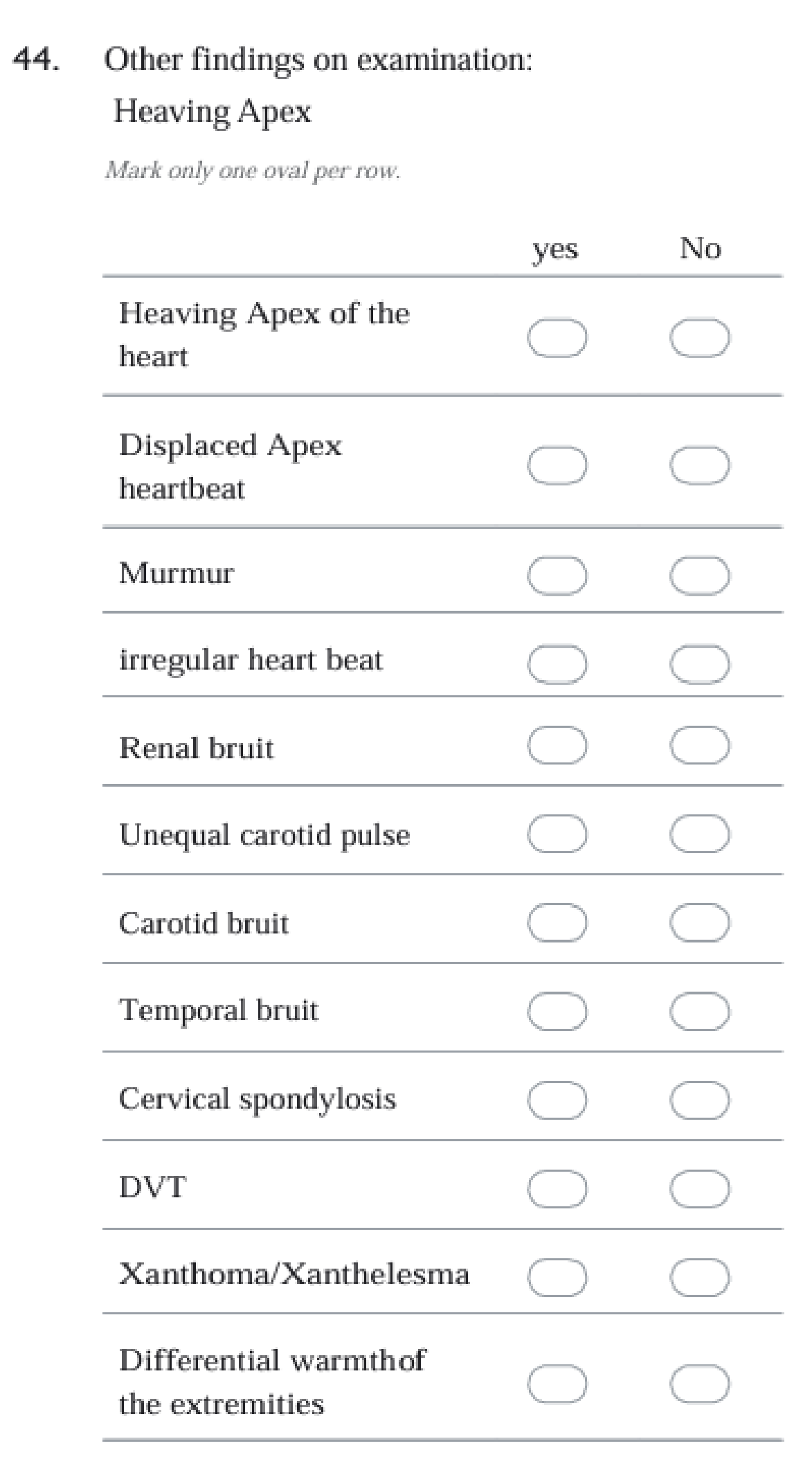

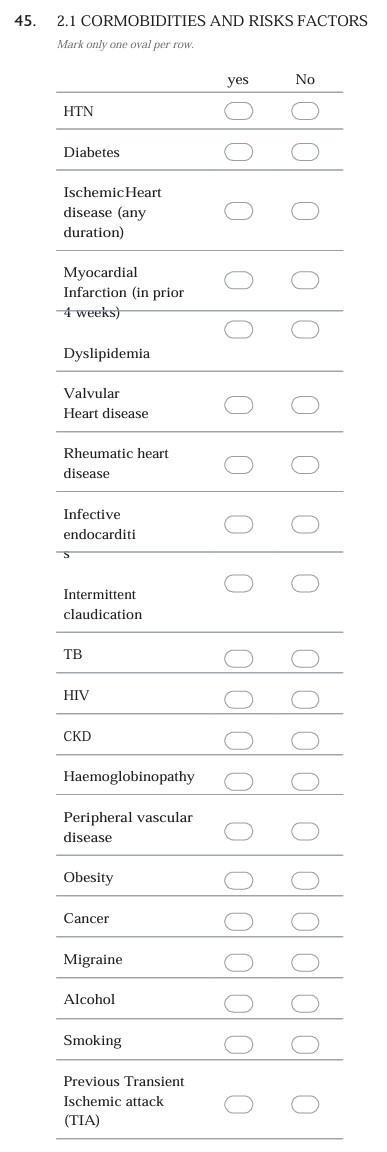


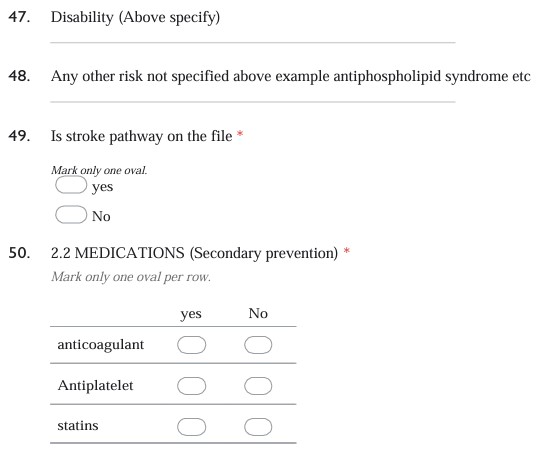


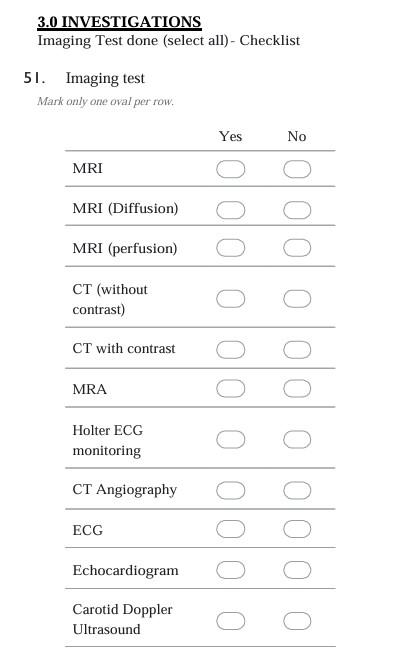


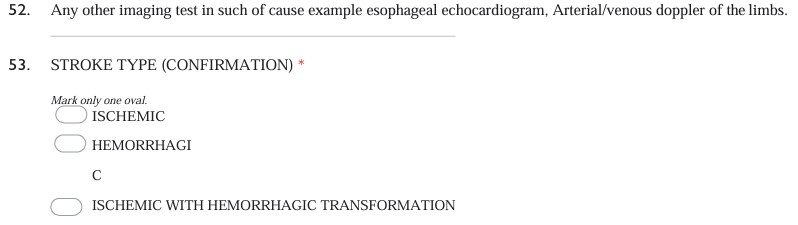


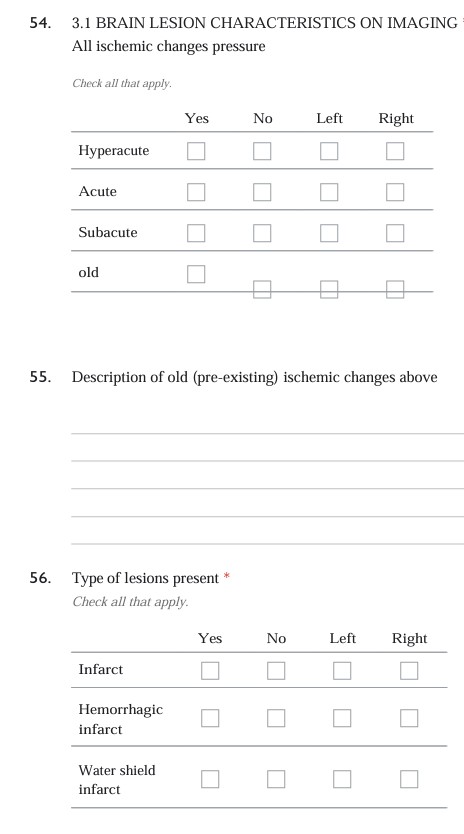


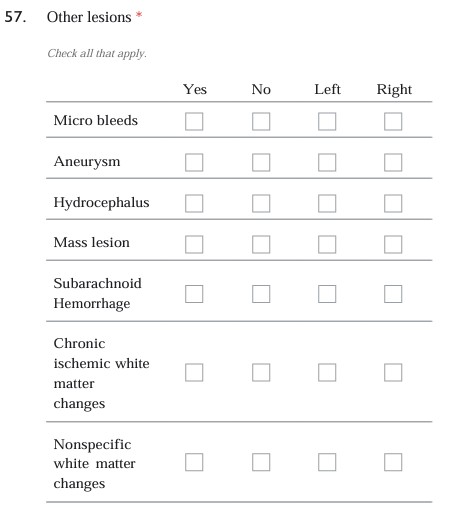


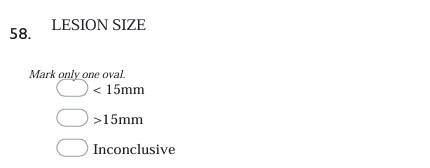


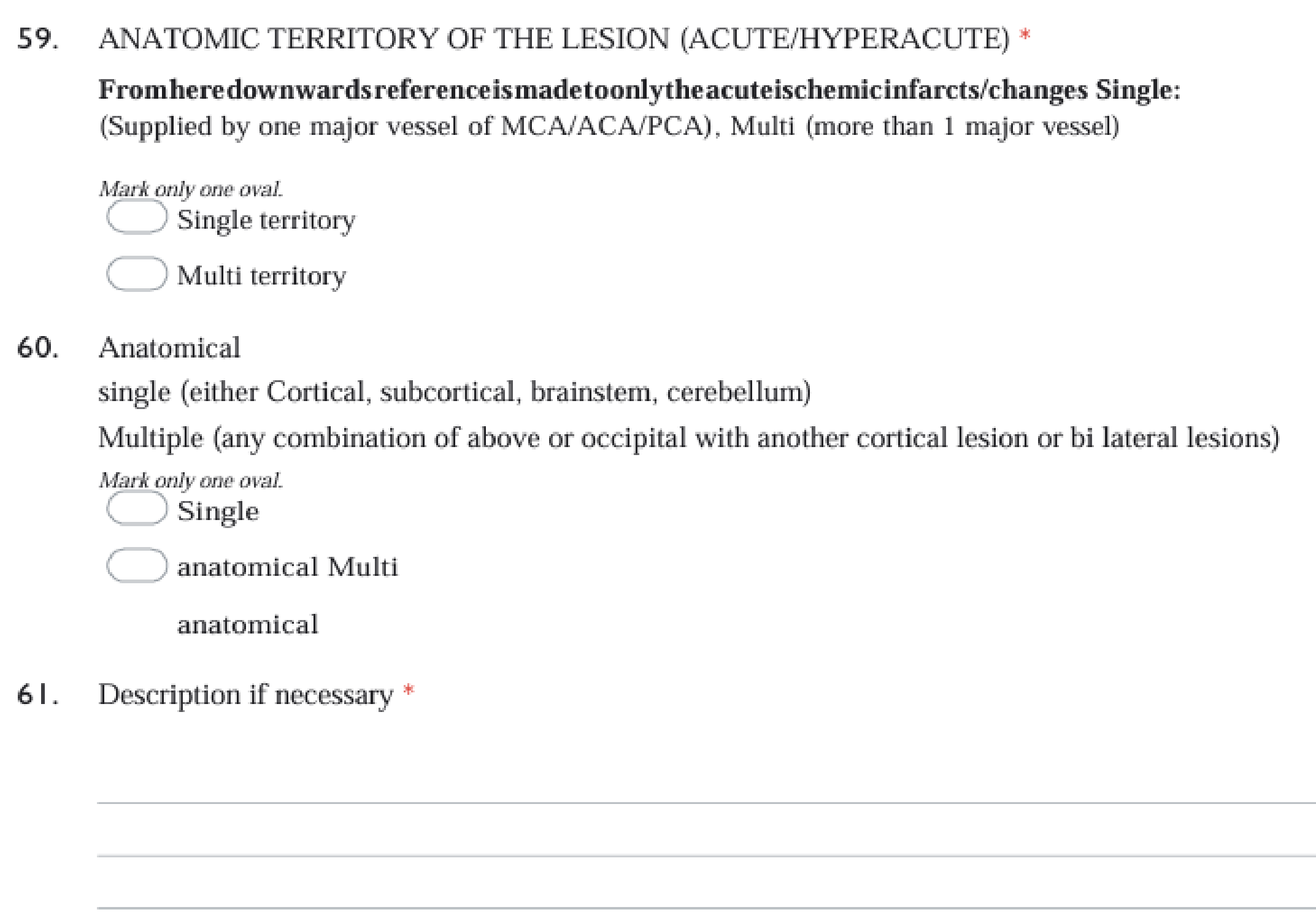


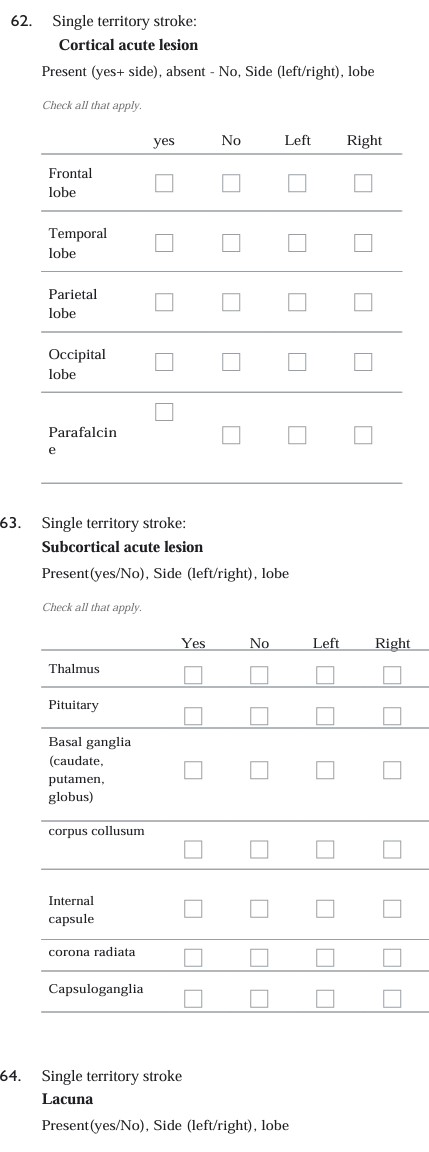


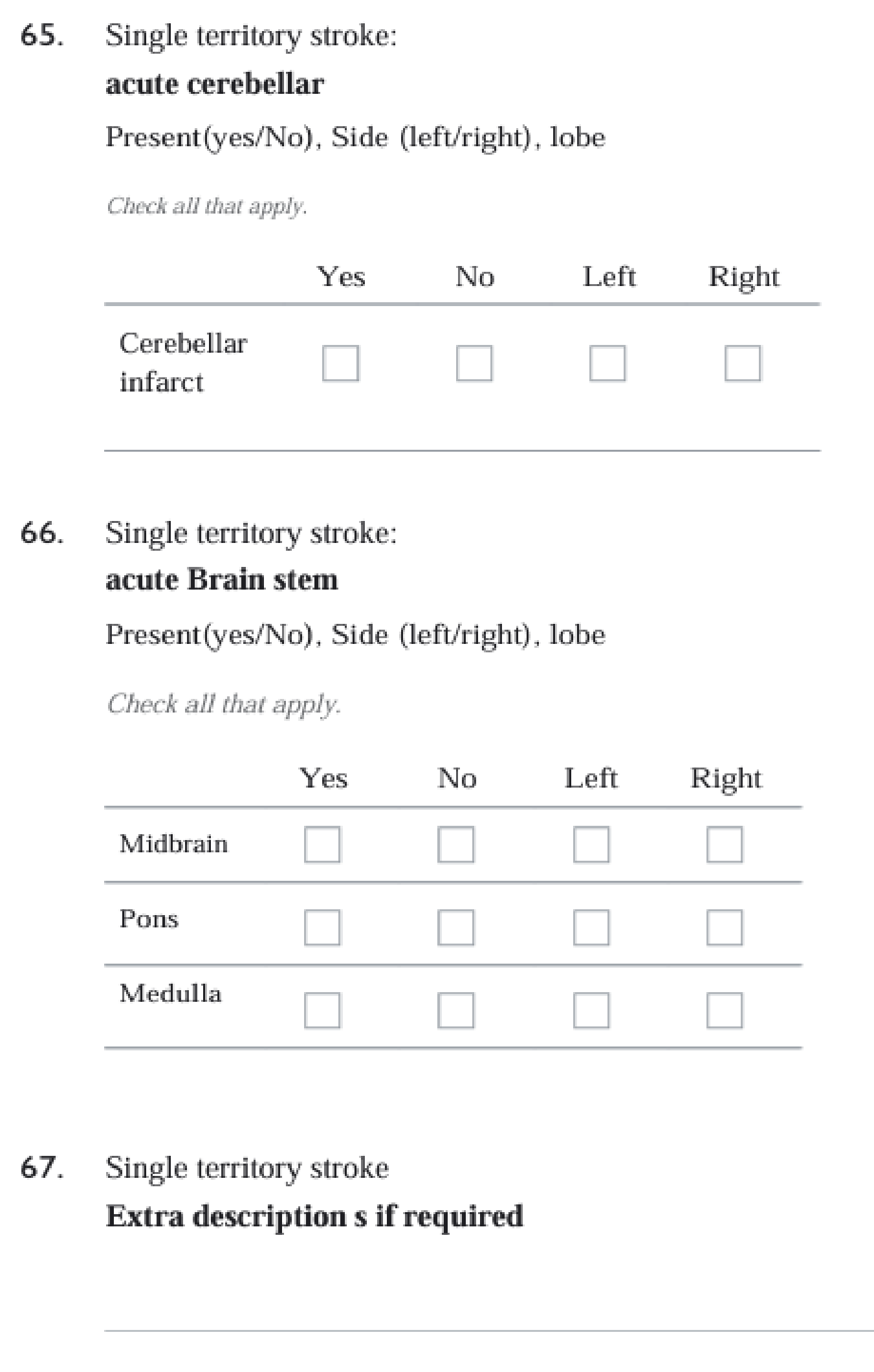


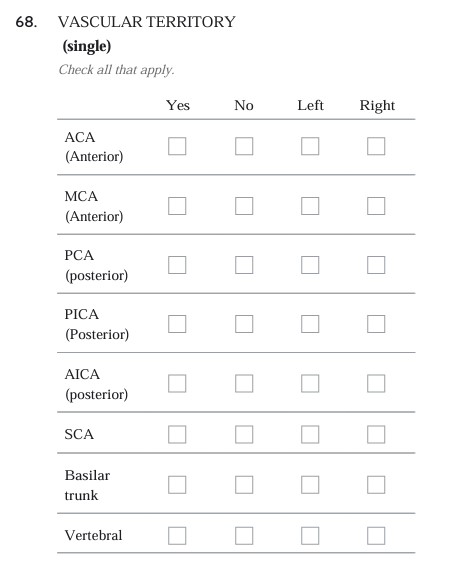


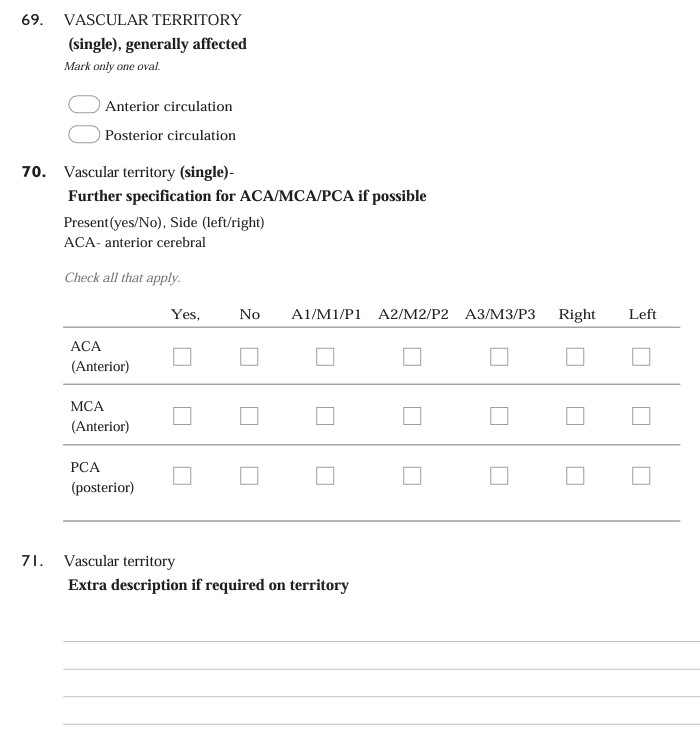


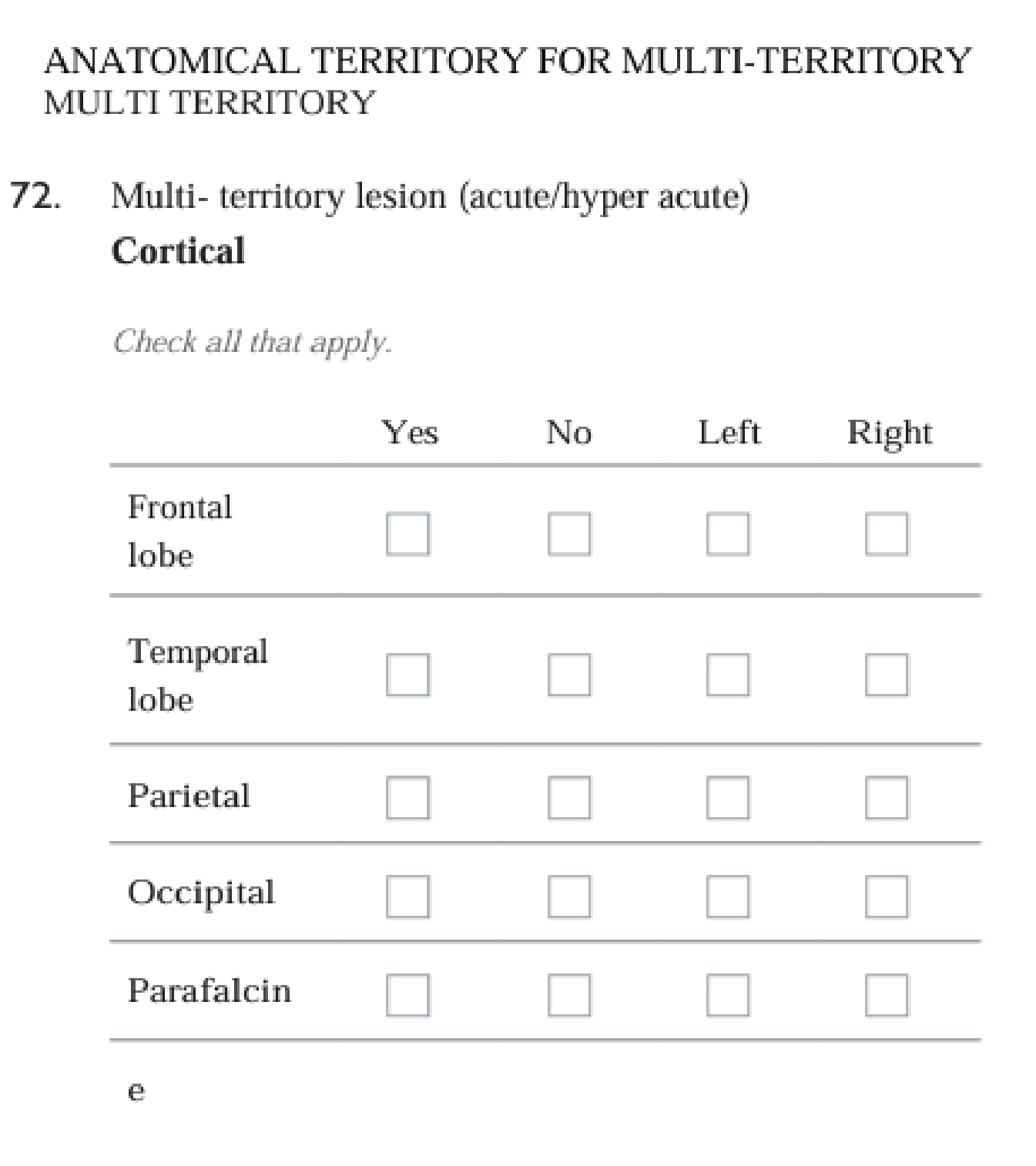

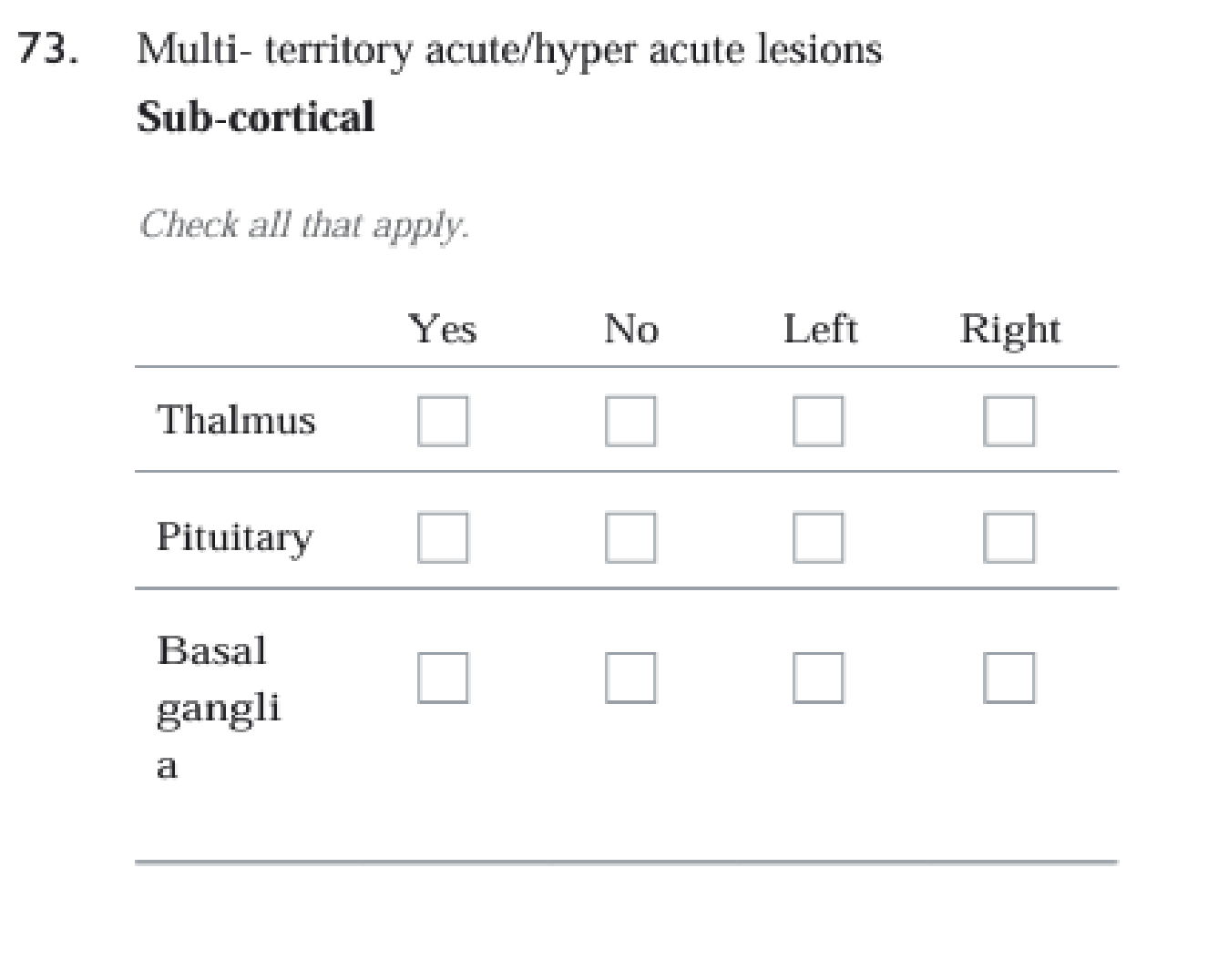


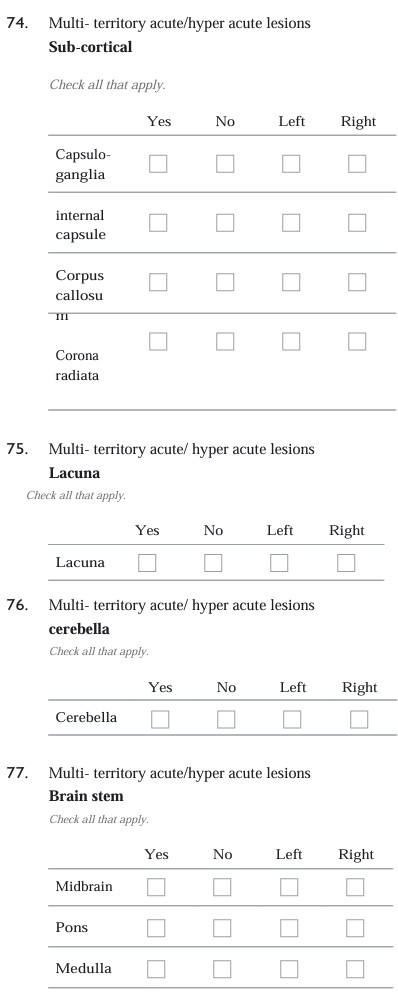


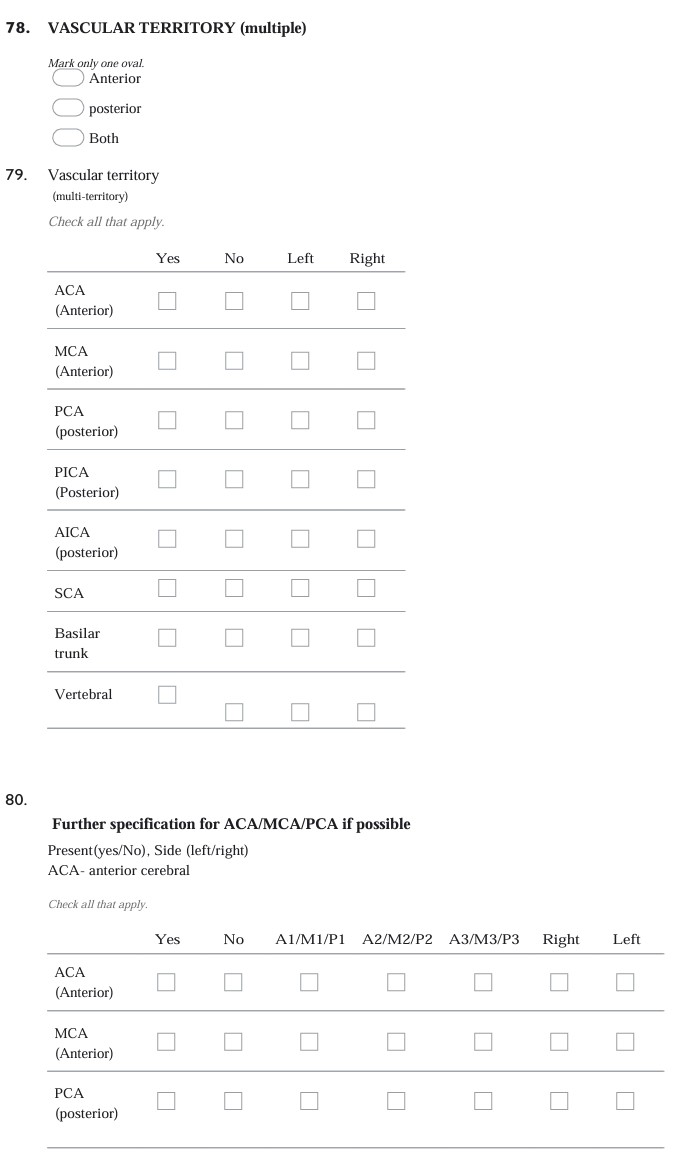

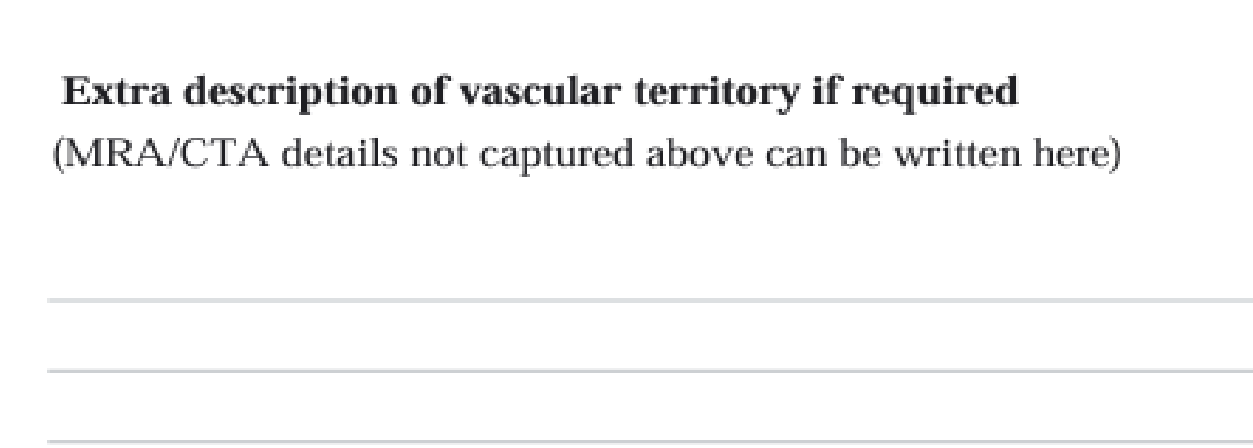


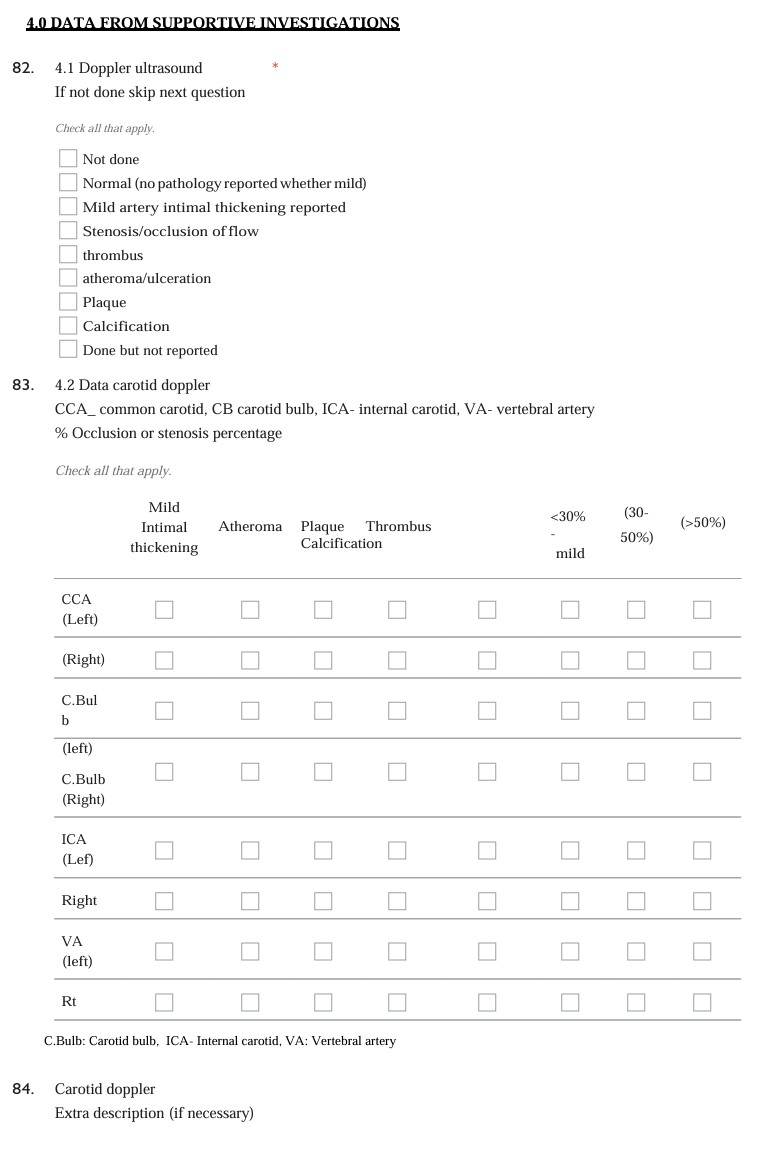


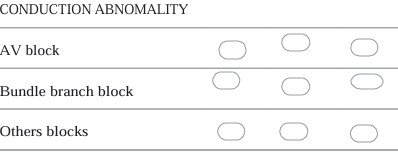


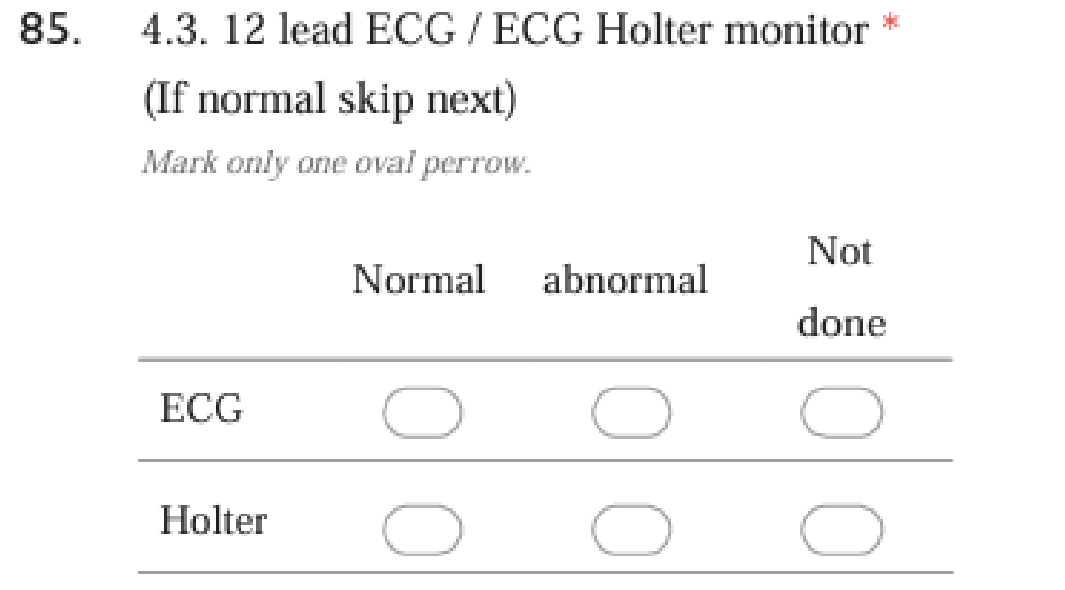

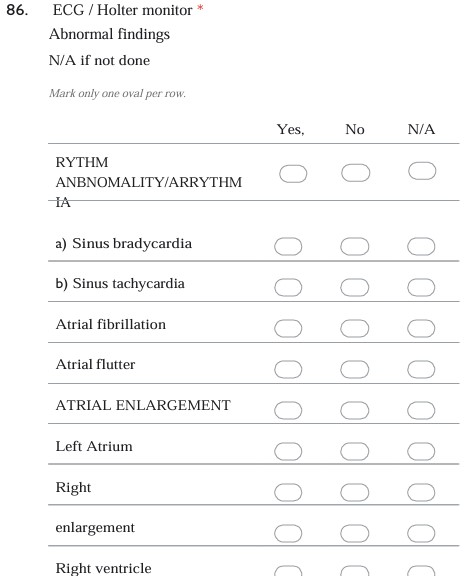

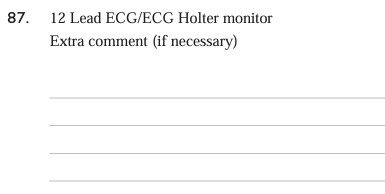

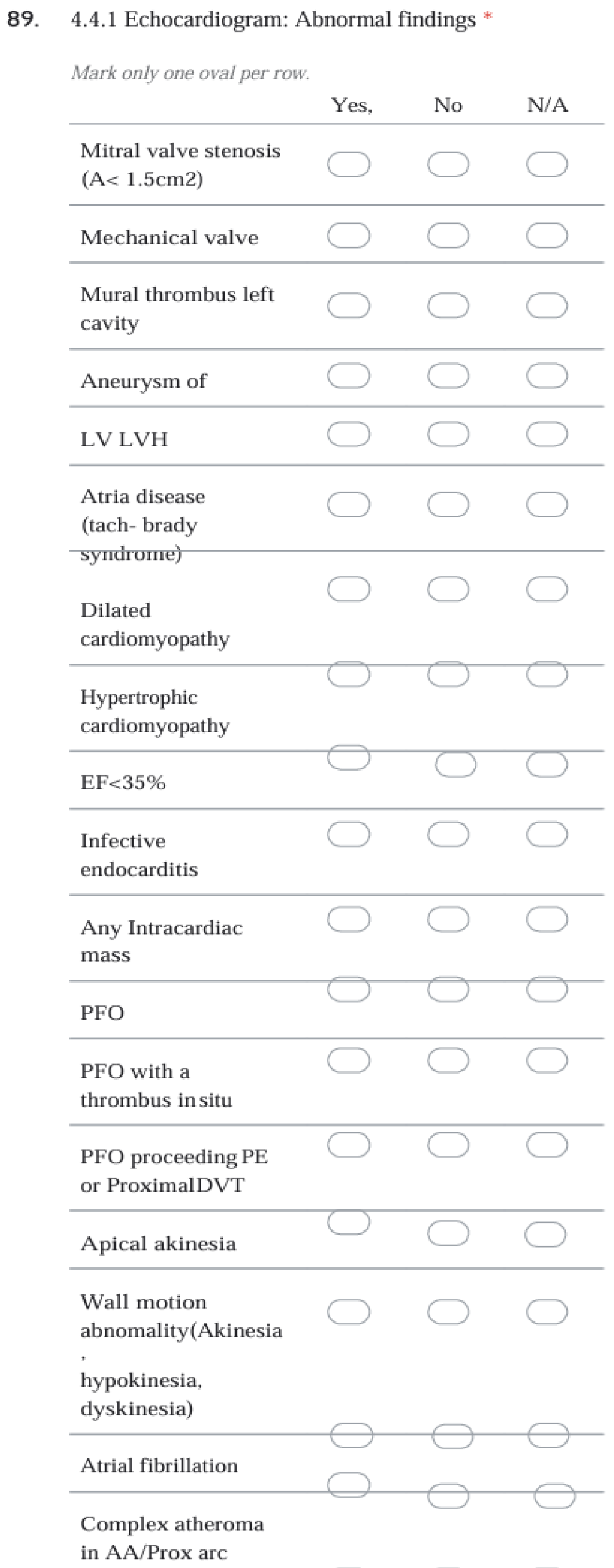

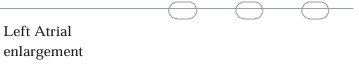

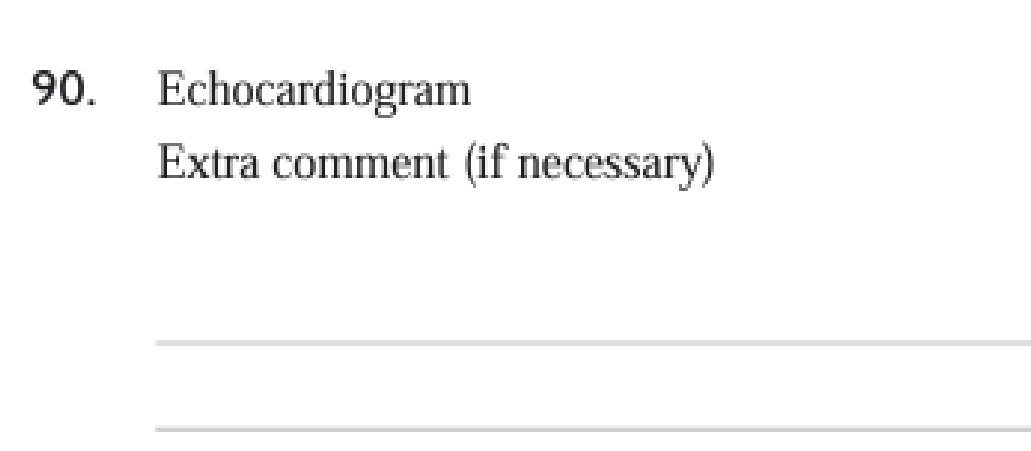

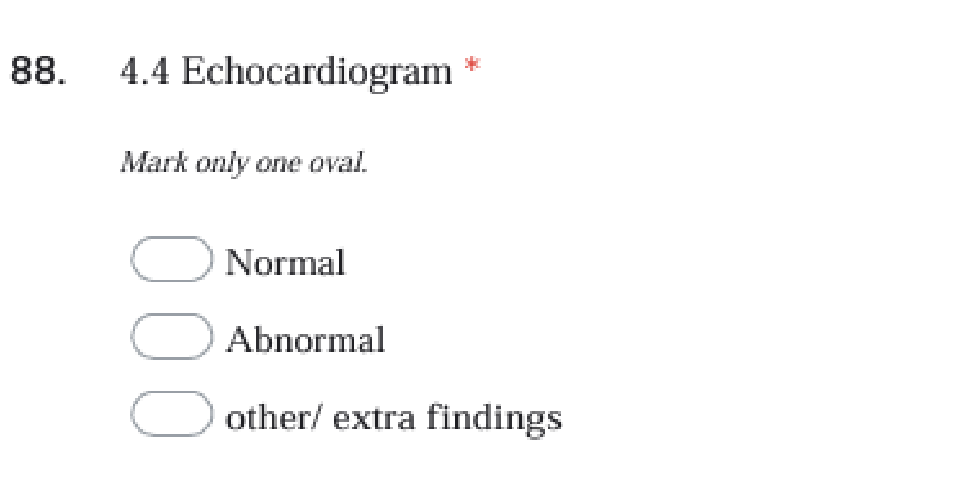

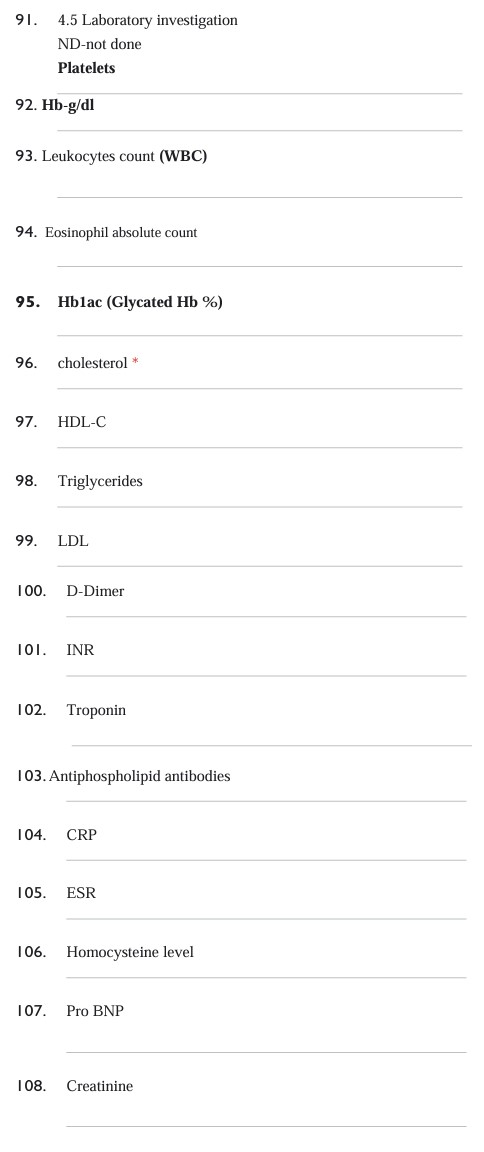

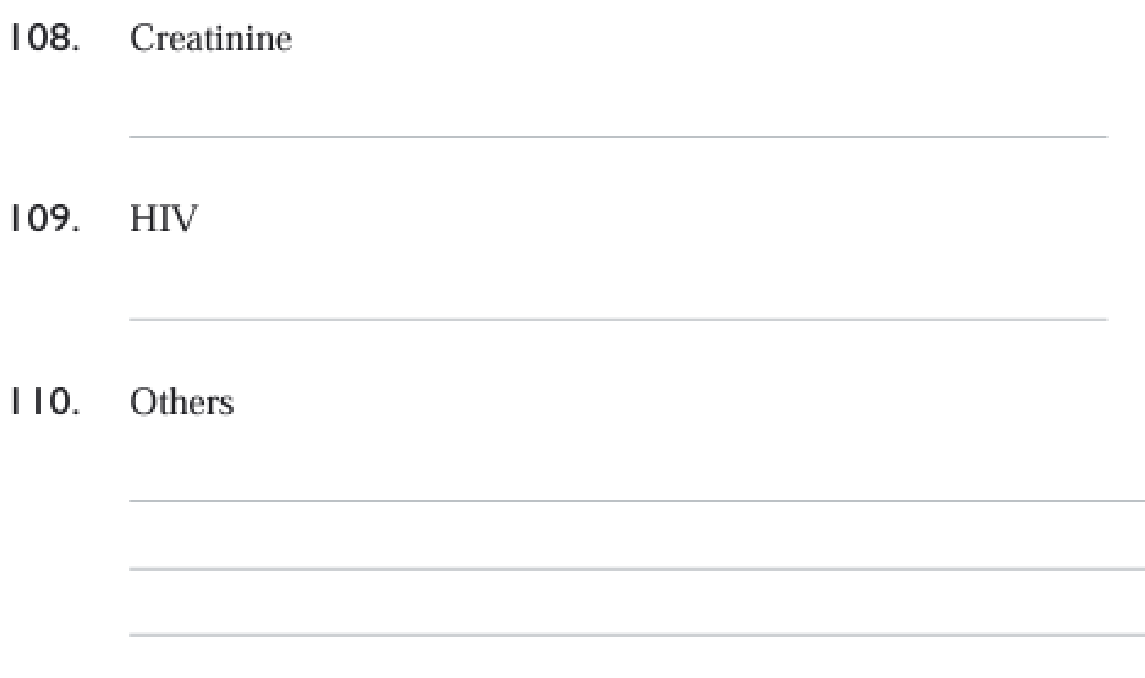


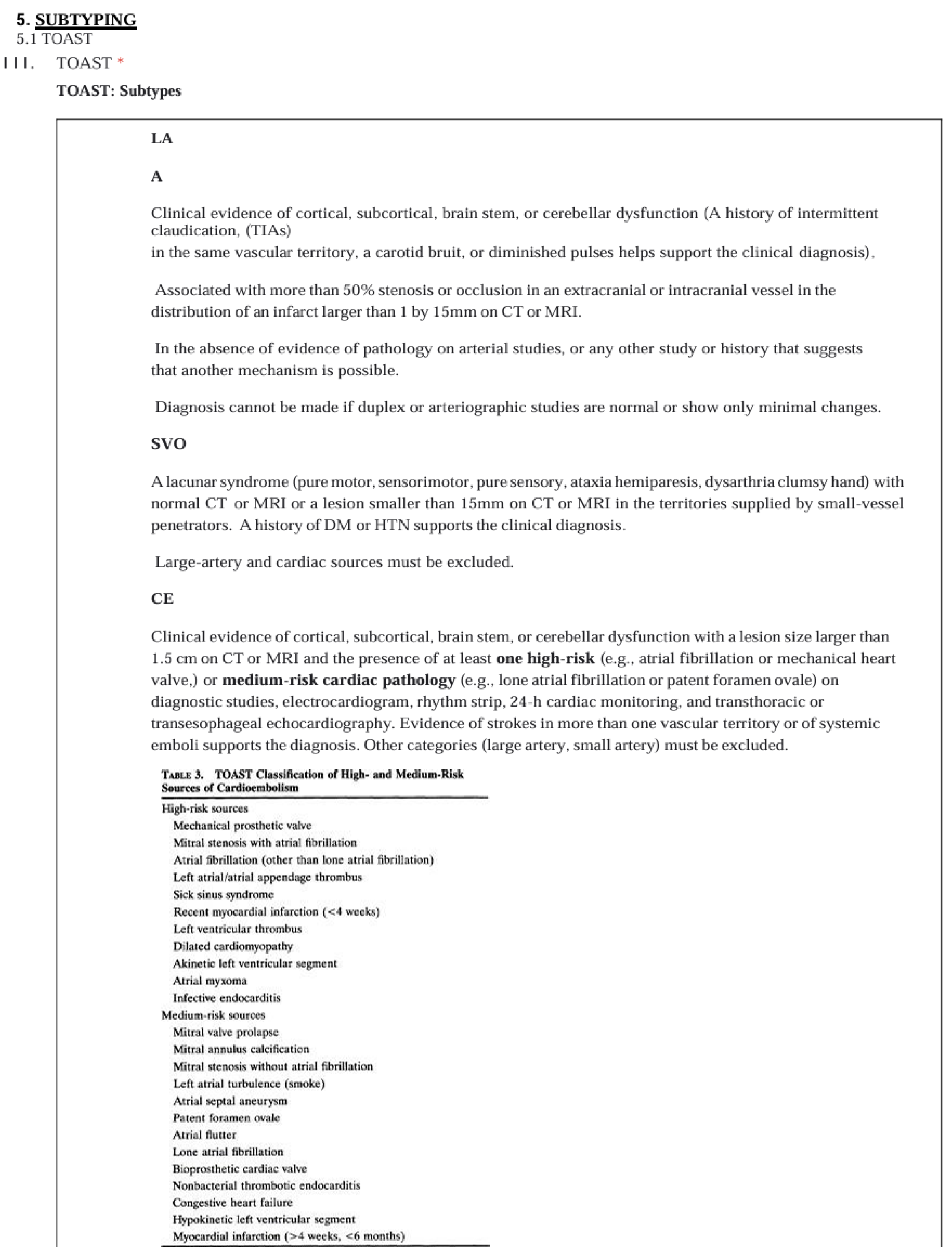


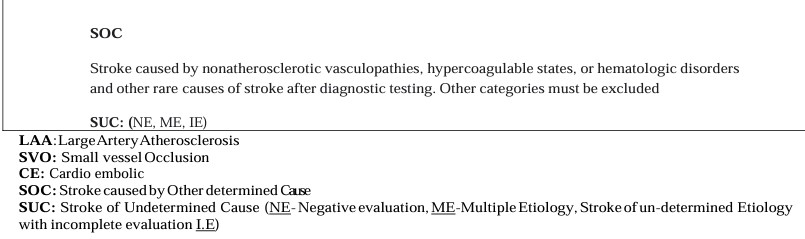

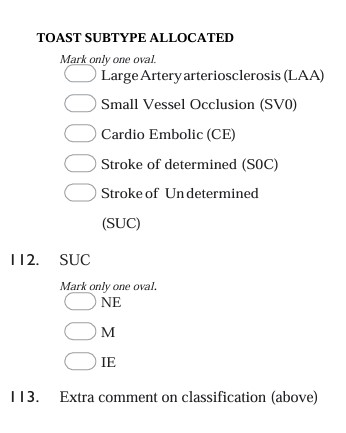


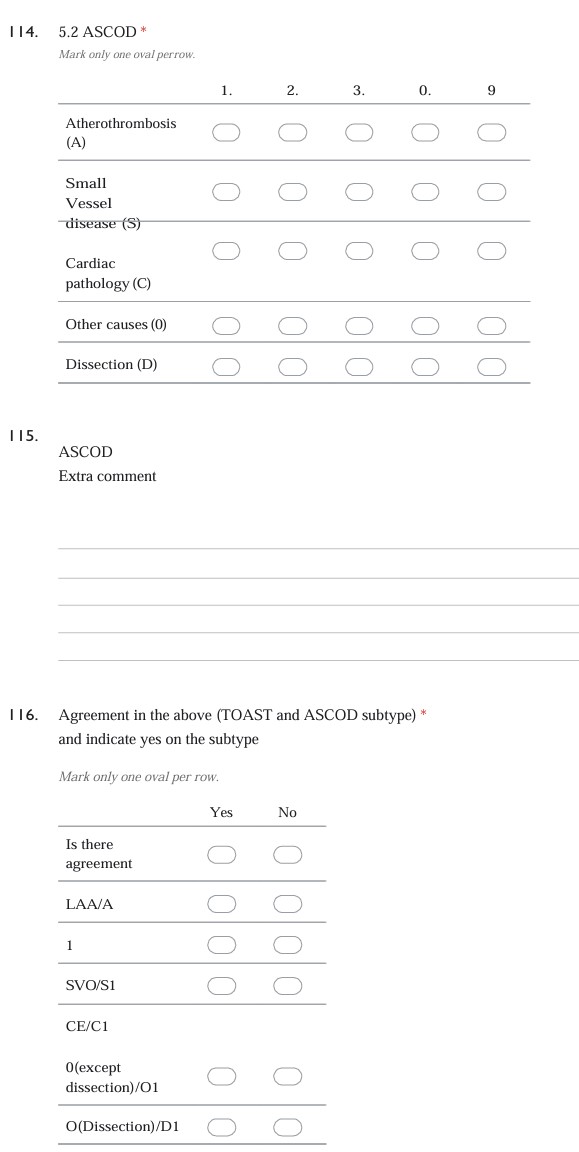

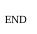

Supplement: Supplementary file 1 [file Supplementary_file_1.docx]
