# Supplementary material for: A comparative analysis of TOAST and ASCOD criteria in etiologic subtyping of acute ischemic stroke at a tertiary hospital in Tanzania
Source: Front Stroke. 2025 Oct 27;4:1598711. doi: 10.3389/fstro.2025.1598711 (PMC12802723; doi:10.3389/fstro.2025.1598711)
Supplement: Supplementary file 2 [file Supplementary_file_2.pdf]

Results

Descriptives

Descriptives

	TOAST	SUC	ASCOD_C	A12	S12	O12/D12	C12
N	130	48	130	130	130	130	130
Missing	0	82	0	0	0	0	0
Mean			1.08	0.238	0.331	0.0692	0.231
Median			0.00	0.00	0.00	0.00	0.00
Standard deviation			2.03	0.428	0.472	0.255	0.423
Minimum			0	0	0	0	0
Maximum			9	1	1	1	1

Frequencies

Frequencies of TOAST

TOAST	Counts	% of Total	Cumulative %
Cardio Embolic (CE)	11	8.5 %	8.5 %
Small Vessel Occlusion (SV0)	38	29.2 %	37.7 %
Large Artery arteriosclerosis (LAA)	29	22.3 %	60.0 %
Stroke of un determined (SUC)	45	34.6 %	94.6 %
Stroke of determined (S0C)	7	5.4 %	100.0 %

Frequencies of SUC

SUC	Counts	% of Total	Cumulative %
Hypercougulable state Known RA, History DVT and also SVT	1	2.1 %	2.1 %
IE	27	56.3 %	58.3 %
ME	13	27.1 %	85.4 %
Dolichoctasis of VA on image others Microangipoathy0	1	2.1 %	87.5 %
NE	5	10.4 %	97.9 %
TB menginitis	1	2.1 %	100.0 %

Frequencies of ASCOD_C

ASCOD_C	Counts	% of Total	Cumulative %
0	81	62.3 %	62.3 %
1	13	10.0 %	72.3 %
2	17	13.1 %	85.4 %
3	13	10.0 %	95.4 %
9	6	4.6 %	100.0 %

Frequencies of A12

A12	Counts	% of Total	Cumulative %
0	99	76.2 %	76.2 %
1	31	23.8 %	100.0 %

Frequencies of S12

S12	Counts	% of Total	Cumulative %
0	87	66.9 %	66.9 %
1	43	33.1 %	100.0 %

Frequencies of O12/D12

O12/D12	Counts	% of Total	Cumulative %
0	121	93.1 %	93.1 %
1	9	6.9 %	100.0 %

Frequencies of C12

C12	Counts	% of Total	Cumulative %
0	100	76.9 %	76.9 %
1	30	23.1 %	100.0 %

Descriptives

Descriptives

N
Missing
Mean
Median
Standard deviation
Minimum
Maximum

Proportion Test (2 Outcomes)

Binomial Test

Level	Count	Total	Proportion	p

Descriptives

Descriptives

	ASCOD_A	ASCOD_S	ASCOD_O	ASCOD_D	A123	S123	C123	O123+D123
N	130	130	130	130	130	130	130	130
Missing	0	0	0	0	0	0	0	0
Mean	1.72	1.12	1.73	1.02	0.469	0.562	0.331	0.123
Median	1.00	1.00	0.00	0.00	0.00	1.00	0.00	0.00
Standard deviation	2.52	1.20	3.36	2.81	0.501	0.498	0.472	0.330
Minimum	0	0	0	0	0	0	0	0
Maximum	9	3	9	9	1	1	1	1

Frequencies

Frequencies of ASCOD_A

ASCOD_A	Counts	% of Total	Cumulative %
0	58	44.6 %	44.6 %
1	28	21.5 %	66.2 %
2	3	2.3 %	68.5 %
3	30	23.1 %	91.5 %
9	11	8.5 %	100.0 %

Frequencies of ASCOD_S

ASCOD_S	Counts	% of Total	Cumulative %
0	57	43.8 %	43.8 %
1	31	23.8 %	67.7 %
2	12	9.2 %	76.9 %
3	30	23.1 %	100.0 %

Frequencies of ASCOD_O

ASCOD_O	Counts	% of Total	Cumulative %
0	93	71.5 %	71.5 %
1	9	6.9 %	78.5 %
3	6	4.6 %	83.1 %
9	22	16.9 %	100.0 %

Frequencies of ASCOD_D

ASCOD_D	Counts	% of Total	Cumulative %
0	114	87.7 %	87.7 %
3	2	1.5 %	89.2 %
9	14	10.8 %	100.0 %

Frequencies of A123

A123	Counts	% of Total	Cumulative %
0	69	53.1 %	53.1 %
1	61	46.9 %	100.0 %

Frequencies of S123

S123	Counts	% of Total	Cumulative %
0	57	43.8 %	43.8 %
1	73	56.2 %	100.0 %

Frequencies of C123

C123	Counts	% of Total	Cumulative %
0	87	66.9 %	66.9 %
1	43	33.1 %	100.0 %

Frequencies of O123+D123

O123+D123	Counts	% of Total	Cumulative %
0	114	87.7 %	87.7 %
1	16	12.3 %	100.0 %

Proportion Test (2 Outcomes)

Binomial Test

	Level	Count	Total	Proportion	p
A12	0	99	130	0.762	< .001
	1	31	130	0.238	< .001
S12	0	87	130	0.669	< .001
	1	43	130	0.331	< .001
C12	0	100	130	0.769	< .001
	1	30	130	0.231	< .001
O12/D12	0	121	130	0.931	< .001
	1	9	130	0.069	< .001

Note. H_a is proportion ≠ 0.5

Interrater Reliability

Interrater Reliability

Method	Cohen's Kappa for 2 Raters (Weights: unweighted)
Subjects	130
Raters	2
Agreement %	95
Kappa	0.843
z	9.61
p-value	< .001

	LAA_TOAST	A1	n
1	0	0	98
2	0	1	3
3	1	0	4
4	1	1	25

Table

```
          A1
LAA_TOAST  0  1
         0 98  3
         1  4 25
```

[3] [4] [5]

Paired Samples Contingency Tables

Contingency Tables

S123		A1 0	A1 1	Total
0	Count	39	18	57
	% within row	68.4 %	31.6 %	
1	Count	63	10	73
	% within row	86.3 %	13.7 %	
Total	Count	102	28	130
	% within row	78.5 %	21.5 %	

McNemar Test

	Value	df	p
χ^2	25.0	1	< .001
N	130		

Proportion Test (2 Outcomes)

Binomial Test

	Level	Count	Total	Proportion	p

Proportion Test (2 Outcomes)

Binomial Test

	Level	Count	Total	Proportion	p
A123	0	69	130	0.531	0.539
	1	61	130	0.469	0.539
S123	0	57	130	0.438	0.188
	1	73	130	0.562	0.188
C123	0	87	130	0.669	< .001
	1	43	130	0.331	< .001
O123	0	115	130	0.885	< .001
	1	15	130	0.115	< .001
D123	0	128	130	0.985	< .001
	1	2	130	0.015	< .001

Note. H_a is proportion ≠ 0.5

Interrater Reliability

Interrater Reliability

Method	Cohen's Kappa for 2 Raters (Weights: unweighted)
Subjects	130
Raters	2
Agreement %	95
Kappa	0.843
z	9.61
p-value	< .001

	LAA_TOAST	A1	n
1	0	0	98
2	0	1	3
3	1	0	4
4	1	1	25

Table

```
          A1
LAA_TOAST  0  1
         0 98  3
         1  4 25
```

[3] [4] [5]

Paired Samples Contingency Tables

Contingency Tables

		A1		
LAA_TOAST		0	1	Total
0	Count	98	3	101
	% within row	97.0 %	3.0 %	
1	Count	4	25	29
	% within row	13.8 %	86.2 %	
Total	Count	102	28	130
	% within row	78.5 %	21.5 %	

McNemar Test

	Value	df	p
χ^2	0.143	1	0.705
N	130		

Interrater Reliability

Interrater Reliability

Method	Cohen's Kappa for 2 Raters (Weights: unweighted)
Subjects	130
Raters	2
Agreement %	94
Kappa	0.827
z	9.43
p-value	< .001

[3] [4] [5]

Paired Samples Contingency Tables

Contingency Tables

	A12		
LAA_TOAST	0	1	Total
0	96	5	101
1	3	26	29
Total	99	31	130

McNemar Test

	Value	df	p
χ^2	0.500	1	0.480
χ^2 continuity correction	0.125	1	0.724
N	130		

Interrater Reliability

Interrater Reliability

Method	.
Subjects	.
Raters	.
Agreement %	.
Kappa	.
z	.
p-value	.

[3] [4] [5]

Reliability Analysis

Scale Reliability Statistics

scale

[6]

Paired Samples Contingency Tables

Contingency Tables

		A12		
LAA_TOAST		0	1	Total
0	Count	96	5	101
	% within row	95.0 %	5.0 %	
1	Count	3	26	29
	% within row	10.3 %	89.7 %	
Total	Count	99	31	130
	% within row	76.2 %	23.8 %	

McNemar Test

	Value	df	p
χ^2	0.500	1	0.480
N	130		

Interrater Reliability

Interrater Reliability

Method	.
Subjects	.
Raters	.
Agreement %	.
Kappa	.
z	.
p-value	.

[3] [4] [5]

Interrater Reliability

Interrater Reliability

Method	.
Subjects	.
Raters	.
Agreement %	.
Kappa	.
z	.
p-value	.

[3] [4] [5]

Interrater Reliability

Interrater Reliability

Method	.
Subjects	.
Raters	.
Agreement %	.
Kappa	.
z	.
p-value	.

[3] [4] [5]

Interrater Reliability

Interrater Reliability

Method	.
Subjects	.
Raters	.
Agreement %	.
Kappa	.
z	.
p-value	.

Table

[3] [4] [5]

Interrater Reliability

Interrater Reliability

Method	Cohen's Kappa for 2 Raters (Weights: unweighted)
Subjects	130
Raters	2
Agreement %	90
Kappa	0.744
z	8.57
p-value	< .001

	SVO_TOAST	S1	n
1	0	0	89
2	0	1	3
3	1	0	10
4	1	1	28

Table

```
         S1
SVO_TOAST  0  1
        0 89  3
        1 10 28
```

[3] [4] [5]

Paired Samples Contingency Tables

Contingency Tables

	S1		
**SVO_TOAST**	**0**	**1**	**Total**
0	89	3	92
1	10	28	38
Total	99	31	130

McNemar Test

	Value	df	p
χ^2	3.77	1	0.052
N	130		

Interrater Reliability

Interrater Reliability

Method	Cohen's Kappa for 2 Raters (Weights: unweighted)
Subjects	130
Raters	2
Agreement %	95
Kappa	0.725
z	8.30
p-value	< .001

[3] [4] [5]

Paired Samples Contingency Tables

Contingency Tables

	C1		
CE_TOAST	0	1	Total
0	115	4	119
1	2	9	11
Total	117	13	130

McNemar Test

	Value	df	p
χ^2	0.667	1	0.414
N	130		

Interrater Reliability

Interrater Reliability

Method	Cohen's Kappa for 2 Raters (Weights: unweighted)
Subjects	130
Raters	2
Agreement %	95
Kappa	0.601
z	6.91
p-value	< .001

	O_TOAST (2)	O1/D1	n
1	0	0	119
2	0	1	4
3	1	0	2
4	1	1	5

Table

```
            O1/D1
O_TOAST (2)    0    1
          0 119    4
          1    2    5
```

[3] [4] [5]

Paired Samples Contingency Tables

Contingency Tables

O_TOAST (2)		O1/D1 0	O1/D1 1	Total
0	Count	119	4	123
	% within row	96.7 %	3.3 %	
1	Count	2	5	7
	% within row	28.6 %	71.4 %	
Total	Count	121	9	130
	% within row	93.1 %	6.9 %	

McNemar Test

	Value	df	p
χ^2	0.667	1	0.414
N	130		

Proportion Test (2 Outcomes)

Binomial Test

	Level	Count	Total	Proportion	p

Proportion Test (2 Outcomes)

Binomial Test

	Level	Count	Total	Proportion	p
ASCOX	0	67	130	0.515	0.793
	1	63	130	0.485	0.793
ASCOY	0	80	130	0.615	0.011
	1	50	130	0.385	0.011

Note. H_a is proportion ≠ 0.5

Interrater Reliability

Interrater Reliability

Method	Cohen's Kappa for 2 Raters (Weights: unweighted)
Subjects	48
Raters	2
Agreement %	Please check the data. It seems that observers do not agree on any cases
Kappa	0.00
z	NaN
p-value	NaN

	SUC	ASCOX	n
1	Hypercougulable state Known RA, History DVT and also SVT	0	1
2	IE	0	2
3	IE	1	25
4	ME	0	7
5	ME	1	6
6	Dolichoctasis of VA on image others Microangipoathy0	0	1
7	NE	1	5
8	TB menginitis	0	1
9		0	55
10		1	27

Table

```
                                                          ASCOX
SUC                                                         0  1
  Hypercougulable state Known RA, History DVT and also SVT  1  0
  IE                                                        2 25
  ME                                                        7  6
  Dolichoctasis of VA on image others Microangipoathy0      1  0
  NE                                                        0  5
  TB menginitis                                             1  0
```

[3] [4] [5]

Descriptives

Descriptives

	SUC(NMI)
N	45
Missing	85
Mean	
Median	
Standard deviation	
Minimum	
Maximum	

Frequencies

Frequencies of SUC(NMI)

SUC(NMI)	Counts	% of Total	Cumulative %
IE	27	60.0 %	60.0 %
ME	13	28.9 %	88.9 %
NE	5	11.1 %	100.0 %

Paired Samples Contingency Tables

Contingency Tables

		S12		
SVO_TOAST		0	1	Total
0	Count	86	6	92
	% within row	93.5 %	6.5 %	
1	Count	1	37	38
	% within row	2.6 %	97.4 %	
Total	Count	87	43	130
	% within row	66.9 %	33.1 %	

McNemar Test

	Value	df	p
χ^2	3.57	1	0.059
N	130		

Paired Samples Contingency Tables

Contingency Tables

	C12		
CE_TOAST	0	1	Total
0	100	19	119
1	0	11	11
Total	100	30	130

McNemar Test

	Value	df	p
χ^2	19.0	1	< .001
χ^2 continuity correction	17.1	1	< .001
N	130		

Paired Samples Contingency Tables

Contingency Tables

	O12/D12		
O_TOAST (2)	0	1	Total
0	119	4	123
1	2	5	7
Total	121	9	130

McNemar Test

	Value	df	p
χ^2	0.667	1	0.414
N	130		

Interrater Reliability

Interrater Reliability

Method	Cohen's Kappa for 2 Raters (Weights: unweighted)
Subjects	130
Raters	2
Agreement %	94
Kappa	0.827
z	9.43
p-value	< .001

	LAA_TOAST	A12	n
1	0	0	96
2	0	1	5
3	1	0	3
4	1	1	26

Table

```
          A12
LAA_TOAST  0  1
        0 96  5
        1  3 26
```

[3] [4] [5]

Interrater Reliability

Interrater Reliability

Method	.
Subjects	.
Raters	.
Agreement %	.
Kappa	.
z	.
p-value	.

[3] [4] [5]

Interrater Reliability

Interrater Reliability

Method	Cohen's Kappa for 2 Raters (Weights: unweighted)
Subjects	130
Raters	2
Agreement %	95
Kappa	0.875
z	10.0
p-value	< .001

	SVO_TOAST	S12	n
1	0	0	86
2	0	1	6
3	1	0	1
4	1	1	37

Table

```
         S12
SVO_TOAST  0  1
        0 86  6
        1  1 37
```

[3] [4] [5]

Interrater Reliability

Interrater Reliability

Method	Cohen's Kappa for 2 Raters (Weights: unweighted)
Subjects	130
Raters	2
Agreement %	95
Kappa	0.601
z	6.91
p-value	< .001

	O_TOAST (2)	O12/D12	n
1	0	0	119
2	0	1	4
3	1	0	2
4	1	1	5

Table

```
              O12/D12
O_TOAST (2)    0    1
             0 119    4
             1   2    5
```

[3] [4] [5]

Interrater Reliability

Interrater Reliability

Method	Fleiss' Kappa for m Raters
Subjects	130
Raters	3
Agreement %	72
Kappa	0.151
z	2.99
p-value	0.003

	CE_TOAST	C12	O12/D12	n
1	0	0	0	93
2	0	0	1	7
3	0	1	0	17
4	0	1	1	2
5	1	1	0	11

Table

```
, , O12/D12 = 0

        C12
CE_TOAST  0  1
       0 93 17
       1  0 11

, , O12/D12 = 1

        C12
CE_TOAST  0  1
       0  7  2
       1  0  0
```

[3] [4] [5]

Proportion Test (2 Outcomes)

Binomial Test

Level	Count	Total	Proportion	p

Descriptives

Descriptives

	Demographics b) Residence
N	130
Missing	0
Mean	
Median	
Standard deviation	
Minimum	
Maximum	

Frequencies

Frequencies of Demographics b) Residence

Demographics b) Residence	Counts	% of Total	Cumulative %
Dar es Salaam	98	75.4 %	75.4 %
Other	16	12.3 %	87.7 %
Comoro	16	12.3 %	100.0 %

Descriptives

Descriptives

	Other residence (specify)	Who was the primary caring specialist
N	14	130
Missing	116	0
Mean		
Median		
Standard deviation		
Minimum		
Maximum		

Frequencies

Frequencies of Other residence (specify)

Other residence (specify)	Counts	% of Total	Cumulative %
Moshi	1	7.1 %	7.1 %
Tanga	3	21.4 %	28.6 %
Arusha	1	7.1 %	35.7 %
Mwanza	1	7.1 %	42.9 %
Chinese	1	7.1 %	50.0 %
Indonesia	1	7.1 %	57.1 %
Kigoma	1	7.1 %	64.3 %
Bukoba	2	14.3 %	78.6 %
Nairobi- kenya	1	7.1 %	85.7 %
Mbeya	1	7.1 %	92.9 %
Belgium	1	7.1 %	100.0 %

Frequencies of Who was the primary caring specialist

Who was the primary caring specialist	Counts	% of Total	Cumulative %
Neurologist	104	80.0 %	80.0 %
Both above (but neurologist just consulted and not primary)	4	3.1 %	83.1 %
Non neurologist physician only	22	16.9 %	100.0 %

Descriptives

Descriptives

	Leukocytes_count	Eosonophil_absolute_count	Platelets
N	130	130	130
Missing	0	0	0
Mean	7.80	0.171	267
Median	6.94	0.125	248
Standard deviation	4.45	0.245	120
Minimum	0.00	0.00	0
Maximum	36.5	2.40	968

Descriptives

Descriptives

N
Missing
Mean
Median
Standard deviation
Minimum
Maximum

Proportion Test (2 Outcomes)

Binomial Test

	Level	Count	Total	Proportion	p

Descriptives

Descriptives

N
Missing
Mean
Median
Standard deviation
Minimum
Maximum

Descriptives

Descriptives

	WBC_ count	Eosonophil_absolute_count2
N	130	130
Missing	0	0
Mean		
Median		
Standard deviation		
Minimum		
Maximum		

Frequencies

Frequencies of WBC_ count

WBC_ count	Counts	% of Total	Cumulative %
1	126	96.9 %	96.9 %
0	4	3.1 %	100.0 %

Frequencies of Eosonophil_absolute_count2

Eosonophil_absolute_count2	Counts	% of Total	Cumulative %
1	126	96.9 %	96.9 %
0	4	3.1 %	100.0 %

Proportion Test (2 Outcomes)

Binomial Test

	Level	Count	Total	Proportion	p
WBC_ count	1	126	130	0.969	< .001
	0	4	130	0.031	< .001
Eosonophil_absolute_count2	1	126	130	0.969	< .001
	0	4	130	0.031	< .001
Hb_2	1	126	130	0.969	< .001
	0	4	130	0.031	< .001
PLT%	1	126	130	0.969	< .001
	0	4	130	0.031	< .001
chole2	1	114	130	0.877	< .001
	0	16	130	0.123	< .001
Trig2	1	113	130	0.869	< .001
	0	17	130	0.131	< .001
HDLC2	1	111	130	0.854	< .001
	0	19	130	0.146	< .001
CRP2	1	85	130	0.654	< .001
	0	45	130	0.346	< .001
ESR2	1	19	130	0.146	< .001
	0	111	130	0.854	< .001
PRO B2	1	26	130	0.200	< .001
	0	104	130	0.800	< .001
DDimer	1	29	130	0.223	< .001
	0	101	130	0.777	< .001
Troponin 2	1	77	130	0.592	0.043
	0	53	130	0.408	0.043
Glycated_Hb2	1	103	130	0.792	< .001
	0	27	130	0.208	< .001

Note. H_a is proportion ≠ 0.5

Proportion Test (2 Outcomes)

Argument 'vars' contains 'ESR>20' which is not present in the dataset

Binomial Test

Level	Count	Total	Proportion	p

Proportion Test (2 Outcomes)

Binomial Test

	Level	Count	Total	Proportion	p
Hb>16	1	11	130	0.085	< .001
	0	119	130	0.915	< .001
WBC>12	1	18	130	0.138	< .001
	0	112	130	0.862	< .001
Eosonophillia>0.5	0	127	130	0.977	< .001
	1	3	130	0.023	< .001
Cholesterol>/=5.2	0	87	130	0.669	< .001
	1	43	130	0.331	< .001
Triglyceride>/=1.7	0	93	130	0.715	< .001
	1	37	130	0.285	< .001
HDL_C	0	81	130	0.623	0.006
	1	49	130	0.377	0.006
Didimer>1	0	109	130	0.838	< .001
	1	21	130	0.162	< .001
CRP>50	1	21	130	0.162	< .001
	0	109	130	0.838	< .001
ESR>20	0	116	130	0.892	< .001
	1	14	130	0.108	< .001
PROBNP>400	0	112	130	0.862	< .001
	1	18	130	0.138	< .001
Platelets>/=800	1	1	130	0.008	< .001
	0	129	130	0.992	< .001
Glycated>7	1	34	130	0.262	< .001
	0	96	130	0.738	< .001
Toponin>14	0	90	130	0.692	< .001
	1	40	130	0.308	< .001

Note. H_a is proportion ≠ 0.5

Descriptives

Descriptives

	Others
N	105
Missing	25
Mean	
Median	
Standard deviation	
Minimum	
Maximum	

Frequencies

Frequencies of Others

Others	Counts	% of Total	Cumulative %
CAG due to dilated cardiomyopathy	1	1.0 %	1.0 %
Antithrombin III (low)- 79% C- 96%, S- 94%	1	1.0 %	1.9 %
RA- normal(14)	1	1.0 %	2.9 %
Antiphospholipid antibodies done (Normal levels) CSF for gene xpert and routine	1	1.0 %	3.8 %
000	51	48.6 %	52.4 %
PSA- 4.5	1	1.0 %	53.3 %
uric acid 370	1	1.0 %	54.3 %
Uric acid- 571	1	1.0 %	55.2 %
uric acid- 166.46	1	1.0 %	56.2 %
TSH- 1.30	1	1.0 %	57.1 %
uric- 399.36	1	1.0 %	58.1 %
CKMB-20.5, TSH- 6.78	1	1.0 %	59.0 %
LDH- 336, Ferretin- normal	1	1.0 %	60.0 %
TSH-1.77	1	1.0 %	61.0 %
serum protein electrophoresis	1	1.0 %	61.9 %
Blood culture: gram negative cougulase negative	1	1.0 %	62.9 %
PSA-153.1	1	1.0 %	63.8 %
ANCA- negative P-ANCA -Negative ANA- Negative Syphyllis- Negative	1	1.0 %	64.8 %
Procalcitonin-0.042 TSH-Normal Procalcitonin-9.31	1	1.0 %	65.7 %
Anti thrombin III- low- 73% - Low Protein C- Low (49%) Protein S- Low -63% ANA- Anti Nuclear factor -negative Ant-DNA - negative	1	1.0 %	66.7 %
ANA and antdsDNA- negative Homocysteine levels-1.6 normal	1	1.0 %	67.6 %
dengue + and normal thyroid tests	1	1.0 %	68.6 %
Uric acid 102	1	1.0 %	69.5 %
uric acid 473	1	1.0 %	70.5 %
PROCALCITONIN- 0.83	1	1.0 %	71.4 %
Uric acid -641	1	1.0 %	72.4 %

Frequencies of Others

Others	Counts	% of Total	Cumulative %
LDH-169	1	1.0 %	73.3 %
1.04	1	1.0 %	74.3 %
CKMB- 16.08	1	1.0 %	75.2 %
TSH-2.16	1	1.0 %	76.2 %
TSH-2.31	1	1.0 %	77.1 %
procalcitonin-0.04	1	1.0 %	78.1 %
Procalcitonin-2.2	1	1.0 %	79.0 %
Procalcitonin- 0.1, uric acid- 499.79	1	1.0 %	80.0 %
Procalcitonin- 79.0 Blood culture showing Klebsella Increased D-dimer	1	1.0 %	81.0 %
Uric acid- 497.84	1	1.0 %	81.9 %
ANA-negative P- ANCAnegative C-ANCA-negative	1	1.0 %	82.9 %
TSH- 0.176, Vitamin B12-921 (H) VitaminD	1	1.0 %	83.8 %
C-ANCA P-ANCA Anti Rho- negative	1	1.0 %	84.8 %
ANA- negative	1	1.0 %	85.7 %
ferretin- 321.0	1	1.0 %	86.7 %
ferretin -130	1	1.0 %	87.6 %
ferretin-941, CSF-PCR	1	1.0 %	88.6 %
serum ADA, CD4- 253	1	1.0 %	89.5 %
TSH-1.97	1	1.0 %	90.5 %
Negative gene expert and CSF analysis	1	1.0 %	91.4 %
uric acid- 358	1	1.0 %	92.4 %
CSF positve of TB, Positive gene expert,	1	1.0 %	93.3 %
0000	1	1.0 %	94.3 %
Protein C & S normal, TSH normal Anticardiolipid profile normal(Igm,IgA IGg) Factor 5 normal Homocytein level levels normal	1	1.0 %	95.2 %
Procalcitonin more than 100 LDH 528.42	1	1.0 %	96.2 %
CEA -108, ANA- negative Folic acid level-10.4	1	1.0 %	97.1 %

Frequencies of Others

Others	Counts	% of Total	Cumulative %
Hepatiti B surface sntigen positive	1	1.0 %	98.1 %
lactate 1.64	1	1.0 %	99.0 %
ferretin-1104 LDH-537.26 Procaltonin 5.50	1	1.0 %	100.0 %

Proportion Test (2 Outcomes)

Binomial Test

Level	Count	Total	Proportion	p

Descriptives

Descriptives

N
Missing
Mean
Median
Standard deviation
Minimum
Maximum

Descriptives

Descriptives

	O12	D12
N	130	130
Missing	0	0
Mean	0.0692	0.00
Median	0.00	0.00
Standard deviation	0.255	0.00
Minimum	0	0
Maximum	1	0

Frequencies

Frequencies of O12

O12	Counts	% of Total	Cumulative %
0	121	93.1 %	93.1 %
1	9	6.9 %	100.0 %

Frequencies of D12

D12	Counts	% of Total	Cumulative %
0	130	100.0 %	100.0 %

Proportion Test (2 Outcomes)

Binomial Test

	Level	Count	Total	Proportion	p
b) Presenting symptoms [Vomiting]	No	118	130	0.908	< .001
	Yes	12	130	0.092	< .001
b) Presenting symptoms [Headache]	Yes	28	130	0.215	< .001
	No	102	130	0.785	< .001
b) Presenting symptoms [Disturbed counsciuness]	No	91	129	0.705	< .001
	Yes	38	129	0.295	< .001
b) Presenting symptoms [Neck stiffness]	No	129	130	0.992	< .001
	Yes	1	130	0.008	< .001
b) Presenting symptoms [Ictus occuring during activity]	No	129	129	1.000	< .001
b) Presenting symptoms [Ictus occuring while at rest]	Yes	122	130	0.938	< .001
	No	8	130	0.062	< .001
b) Presenting symptoms [Parasthesia]	Yes	28	130	0.215	< .001
	No	102	130	0.785	< .001
b) Presenting symptoms [Gait distubance]	No	120	130	0.923	< .001
	Yes	10	130	0.077	< .001
b) Presenting symptoms [Vertigo]	No	124	128	0.969	< .001
	Yes	4	128	0.031	< .001
b) Presenting symptoms [Swallowing difficulty]	No	115	127	0.906	< .001
	Yes	12	127	0.094	< .001
Side of Limb weakness [Upper]	No weakness present	24	128	0.188	< .001
	Left	55	128	0.430	0.133
	Right	47	128	0.367	0.003
	Both	2	128	0.016	< .001
Paresthesia (side) [Parashesia]	Not present	103	130	0.792	< .001
	Left	19	130	0.146	< .001
	Right	8	130	0.062	< .001
Facial weakness	Yes	63	130	0.485	0.793
	No	67	130	0.515	0.793
Facial weakness(side) [Mouth deviation away]	No weakness (N/A)	69	128	0.539	0.426
	Left	30	128	0.234	< .001
	Right	29	128	0.227	< .001

Note. H_a is proportion ≠ 0.5

Proportion Test (2 Outcomes)

Variable 'Multi- territory acute/hyper acute lesions Sub-cortical [049-69-46 FRITZ LUDIN]' contains no data

Binomial Test

	Level	Count	Total	Proportion	p
Multi- territory acute/hyper acute lesions Sub-cortical [049-69-46 FRITZ LUDIN]	…	.	.	.	.
	…	.	.	.	.

Note. H_a is proportion ≠ 0.5

Proportion Test (2 Outcomes)

Binomial Test

	Level	Count	Total	Proportion	p
A0S0C0O0D0	0	126	130	0.969	< .001
	1	4	130	0.031	< .001
ASCOZ	0	114	130	0.877	< .001
	1	16	130	0.123	< .001

Note. H_a is proportion ≠ 0.5

Paired Samples Contingency Tables

Contingency Tables

	.		
.	.	.	Total
.	.	.	.
.	.	.	.
Total	.	.	.

McNemar Test

	Value	df	p
χ^2	.	.	.
N	.		

Reliability Analysis

Scale Reliability Statistics

	Cronbach's α
scale	.

[6]

Paired Samples Contingency Tables

Contingency Tables

SUC (2)	ASCOX 0	ASCOX 1	Total
1	9	36	45
0	58	27	85
Total	67	63	130

McNemar Test

	Value	df	p
χ^2	5.15	1	0.023
N	130		

Interrater Reliability

Interrater Reliability

Method	Cohen's Kappa for 2 Raters (Weights: unweighted)
Subjects	130
Raters	2
Agreement %	72
Kappa	0.441
z	5.24
p-value	< .001

	SUC (2)	ASCOX	n
1	1	0	9
2	1	1	36
3	0	0	58
4	0	1	27

Table

```
         ASCOX
SUC (2)    0  1
        1  9 36
        0 58 27
```

[3] [4] [5]

Paired Samples Contingency Tables

Contingency Tables

	ASCOY		
SUC (2)	0	1	Total
1	12	33	45
0	68	17	85
Total	80	50	130

McNemar Test

	Value	df	p
χ^2	12.1	1	< .001
N	130		

Interrater Reliability

Interrater Reliability

Method	Cohen's Kappa for 2 Raters (Weights: unweighted)
Subjects	130
Raters	2
Agreement %	78
Kappa	0.520
z	5.95
p-value	< .001

[3] [4] [5]

Proportion Test (2 Outcomes)

Binomial Test

	Level	Count	Total	Proportion	p
ASCODI	0	130	130	1.000	< .001

Note. H_a is proportion ≠ 0.5

Proportion Test (2 Outcomes)

Binomial Test

	Level	Count	Total	Proportion	p
3.1 BRAIN LESION CHARACTERISTICS ON IMAGING All ischemic changes pressure [Hyperacute]	No	119	130	0.915	< .001
	Yes	1	130	0.008	< .001
	No, Left	2	130	0.015	< .001
	Yes, Right	1	130	0.008	< .001
	Yes, Left	6	130	0.046	< .001
	No, Left, Right	1	130	0.008	< .001
3.1 BRAIN LESION CHARACTERISTICS ON IMAGING All ischemic changes pressure [Subacute]	No	107	130	0.823	< .001
	Yes, Left	7	130	0.054	< .001
	Yes, Left, Right	4	130	0.031	< .001
	Yes, Right	12	130	0.092	< .001
Type of lesions present [Infarct]	Yes, Right	48	130	0.369	0.004
	No	2	130	0.015	< .001
	Yes	7	130	0.054	< .001
	Yes, Left	56	130	0.431	0.136
	Yes, Left, Right	15	130	0.115	< .001
	No, Right	1	130	0.008	< .001
	Yes, No, Left	1	130	0.008	< .001
Other lesions [Micro bleeds]	No	128	130	0.985	< .001
	Yes, Left	1	130	0.008	< .001
	Yes, Left, Right	1	130	0.008	< .001
Other lesions [Non specific white matter changes]	Yes	6	130	0.046	< .001
	No	92	130	0.708	< .001
	Yes, No	1	130	0.008	< .001
	Yes, Left, Right	30	130	0.231	< .001
	Yes, No, Left, Right	1	130	0.008	< .001
Single territory stroke: Cortical acute lesion [Frontal lobe]	No	66	82	0.805	< .001
	yes, Right	8	82	0.098	< .001
	yes, Left	8	82	0.098	< .001

Note. H_a is proportion ≠ 0.5

Proportion Test (2 Outcomes)

Binomial Test

	Level	Count	Total	Proportion	p
Single territory stroke: Cortical acute lesion [Occipital lobe]	No	74	82	0.902	< .001
	yes, Right	3	82	0.037	< .001
	yes, Left	5	82	0.061	< .001
Single territory stroke: Cortical acute lesion [Parietal lobe]	No	61	82	0.744	< .001
	yes, Right	14	82	0.171	< .001
	yes, Left	7	82	0.085	< .001
Single territory stroke: Cortical acute lesion [parafalcine]	No	81	82	0.988	< .001
	yes, Left	1	82	0.012	< .001
Single territory stroke: Cortical acute lesion [Frontal lobe]	No	66	82	0.805	< .001
	yes, Right	8	82	0.098	< .001
	yes, Left	8	82	0.098	< .001
Single territory stroke: Cortical acute lesion [Temporal lobe]	No	68	82	0.829	< .001
	yes, Left	5	82	0.061	< .001
	yes, Right	8	82	0.098	< .001
	Left	1	82	0.012	< .001
Single territory stroke: Subcortical acute lesion [Thalmus]	No	70	81	0.864	< .001
	Yes, Left	8	81	0.099	< .001
	Yes, Right	3	81	0.037	< .001
Single territory stroke: Subcortical acute lesion [Pituitary]	No	81	81	1.000	< .001
Single territory stroke: Subcortical acute lesion [Basal ganglia (caudate, putamen, globus)]	No	64	81	0.790	< .001
	Yes, Right	10	81	0.123	< .001
	Yes, Left	7	81	0.086	< .001
Single territory stroke: Subcortical acute lesion [corpus collusum]	No	77	81	0.951	< .001
	Yes, Left	3	81	0.037	< .001
	Yes, Right	1	81	0.012	< .001
Single territory stroke: Subcortical acute lesion [Internal capsule]	No	76	81	0.938	< .001
	Yes, Left	3	81	0.037	< .001
	Yes, Right	2	81	0.025	< .001
Single territory stroke Lacuna [Lacuna]	Yes, Right	4	79	0.051	< .001
	No	66	79	0.835	< .001
	Yes, Left	9	79	0.114	< .001
Single territory stroke acute cerebellar [Cerebellar infarct]	No	75	79	0.949	< .001
	Yes, Left	3	79	0.038	< .001
	Yes, Right	1	79	0.013	< .001
Single territory stroke acute Brain stem [Midbrain]	No	80	80	1.000	< .001
Single territory stroke acute Brain stem [Pons]	No	76	80	0.950	< .001
	Yes, Left	3	80	0.037	< .001
	Yes, Right	1	80	0.013	< .001
Single territory stroke acute Brain stem [Medulla]	No	75	77	0.974	< .001
	Yes, Left	1	77	0.013	< .001
	Yes, Right	1	77	0.013	< .001

Note. H_a is proportion ≠ 0.5

Binomial Test

	Level	Count	Total	Proportion	p
ANATOMIC TERRITORY OF THE LESION (ACUTE/HYPERACUTE)	Mult-territory	51	130	0.392	0.018
	Single territory	79	130	0.608	0.018

Note. H_a is proportion ≠ 0.5

Proportion Test (2 Outcomes)

Binomial Test

	Level	Count	Total	Proportion	p
LESION SIZE	>15mm	64	108	0.593	0.067
	< 15mm	44	108	0.407	0.067
VASCULAR TERRITORY (multiple)	Both	26	53	0.491	1.000
	Anterior	17	53	0.321	0.013
	posterior	10	53	0.189	< .001
VASCULAR TERRITORY (single) [MCA (Anterior)]	No	13	65	0.200	< .001
	Yes	2	65	0.031	< .001
	Yes, Left	25	65	0.385	0.082
	Yes, Right	25	65	0.385	0.082
VASCULAR TERRITORY (single) [ACA (Anterior)]	No	31	36	0.861	< .001
	Yes, Right	2	36	0.056	< .001
	Yes, Left	3	36	0.083	< .001
Vascular territory (single)- Further specification for ACA/MCA/PCA if possible [ACA (Anterior)]	A2/M2/P2	1	4	0.250	0.625
	Yes, A2/M2/P2, Left	1	4	0.250	0.625
	Yes, A2/M2/P2, Right	1	4	0.250	0.625
	Yes, A2/M2/P2	1	4	0.250	0.625

Note. H_a is proportion ≠ 0.5

Descriptives

Descriptives

N
Missing
Mean
Median
Standard deviation
Minimum
Maximum

Proportion Test (2 Outcomes)

Binomial Test

	Level	Count	Total	Proportion	p
VASCULAR TERRITORY (multiple)	Both	26	53	0.491	1.000
	Anterior	17	53	0.321	0.013
	posterior	10	53	0.189	< .001
Vascular territory (Multi-territory) [ACA (Anterior)]	No	12	21	0.571	0.664
	Yes, Right	5	21	0.238	0.027
	Yes, Left	4	21	0.190	0.007
Vascular territory (Multi-territory) [PCA (posterior)]	Yes, Right	11	30	0.367	0.200
	Yes, Left	9	30	0.300	0.043
	No	7	30	0.233	0.005
	Yes, Left, Right	1	30	0.033	< .001
	Yes	2	30	0.067	< .001
Vascular territory (Multi-territory) [AICA (posterior)]	No	13	16	0.813	0.021
	Yes, Left	3	16	0.188	0.021
Vascular territory (Multi-territory) [MCA (Anterior)]	Yes, Right	21	44	0.477	0.880
	Yes	3	44	0.068	< .001
	Yes, Left	10	44	0.227	< .001
	No	4	44	0.091	< .001
	Yes, Left, Right	6	44	0.136	< .001

Note. H_a is proportion ≠ 0.5

Proportion Test (2 Outcomes)

Binomial Test

	Level	Count	Total	Proportion	p
VASCULAR TERRITORY (single) [ACA (Anterior)]	No	31	36	0.861	< .001
	Yes, Right	2	36	0.056	< .001
	Yes, Left	3	36	0.083	< .001
VASCULAR TERRITORY (single) [PCA (posterior)]	No	27	40	0.675	0.038
	Yes, Left	9	40	0.225	< .001
	Yes	1	40	0.025	< .001
	Yes, Right	3	40	0.075	< .001
VASCULAR TERRITORY (single) [AICA (posterior)]	No	34	34	1.000	< .001
VASCULAR TERRITORY (single) [Basiilar trunk]	No	34	35	0.971	< .001
	Yes, Left, Right	1	35	0.029	< .001
VASCULAR TERRITORY (single) [MCA (Anterior)]	No	13	65	0.200	< .001
	Yes	2	65	0.031	< .001
	Yes, Left	25	65	0.385	0.082
	Yes, Right	25	65	0.385	0.082
VASCULAR TERRITORY (single) [SCA]	No	34	36	0.944	< .001
	Yes, Left	1	36	0.028	< .001
	Yes, Right	1	36	0.028	< .001
VASCULAR TERRITORY (single) [PICA (Posterior)]	No	33	39	0.846	< .001
	Yes, Left	4	39	0.103	< .001
	Yes, Right	2	39	0.051	< .001
VASCULAR TERRITORY (single) [Vertebral]	No	34	35	0.971	< .001
	Yes, Right	1	35	0.029	< .001

Note. H_a is proportion ≠ 0.5

Proportion Test (2 Outcomes)

Binomial Test

Level	Count	Total	Proportion	p

Proportion Test (2 Outcomes)

Binomial Test

Level	Count	Total	Proportion	p

Descriptives

Descriptives

N
Missing
Mean
Median
Standard deviation
Minimum
Maximum

Proportion Test (2 Outcomes)

Binomial Test

	Level	Count	Total	Proportion	p

Proportion Test (2 Outcomes)

Binomial Test

						95% Confidence Interval	
	Level	Count	Total	Proportion	p	Lower	Upper

Proportion Test (2 Outcomes)

Binomial Test

	Level	Count	Total	Proportion	p

Descriptives

Descriptives

N
Missing
Mean
Median
Standard deviation
Minimum
Maximum

Descriptives

Descriptives

	NIHSS score for this stroke
N	125
Missing	5
Mean	
Median	
Standard deviation	
Minimum	
Maximum	

Frequencies

Frequencies of NIHSS score for this stroke

NIHSS score for this stroke	Counts	% of Total	Cumulative %
ND	56	44.8 %	44.8 %
5	5	4.0 %	48.8 %
01	3	2.4 %	51.2 %
04	7	5.6 %	56.8 %
06	4	3.2 %	60.0 %
03	6	4.8 %	64.8 %
2	2	1.6 %	66.4 %
02	7	5.6 %	72.0 %
12	1	0.8 %	72.8 %
10	7	5.6 %	78.4 %
15	1	0.8 %	79.2 %
11	6	4.8 %	84.0 %
0	2	1.6 %	85.6 %
16	1	0.8 %	86.4 %
13	2	1.6 %	88.0 %
05	3	2.4 %	90.4 %
22	1	0.8 %	91.2 %
00	3	2.4 %	93.6 %
08	3	2.4 %	96.0 %
07	1	0.8 %	96.8 %
17	2	1.6 %	98.4 %
4	1	0.8 %	99.2 %
8	1	0.8 %	100.0 %

References

[1] The jamovi project (2023). *jamovi*. (Version 2.4) [Computer Software]. Retrieved from https://www.jamovi.org.

[2] R Core Team (2022). *R: A Language and environment for statistical computing*. (Version 4.1) [Computer software]. Retrieved from https://cran.r-project.org. (R packages retrieved from CRAN snapshot 2023-04-07).

[3] Matthias Gamer, Jim Lemon, Ian Fellows, Puspendra Singh (2019). *Various Coefficients of Interrater Reliability and Agreement.*. [R package]. Retrieved from https://CRAN.R-project.org/package=irr.

[4] (2020). Pathologists should probably forget about kappa. Percent agreement, diagnostic specificity and related metrics provide more clinically applicable measures of interobserver variability. *Annals of Diagnostic Pathology. link, 47*.

[5] Serdar Balci (2022). *ClinicoPath jamovi Module doi:10.5281/zenodo.3997188*. [R package]. Retrieved from https://github.com/sbalci/ClinicoPathJamoviModule. link.

[6] Revelle, W. (2023). *psych: Procedures for Psychological, Psychometric, and Personality Research*. [R package]. Retrieved from https://cran.r-project.org/package=psych.
